# Coordinated modulation of multiple processes through phase variation of a c-di-GMP phosphodiesterase in *Clostridioides difficile*

**DOI:** 10.1371/journal.ppat.1010677

**Published:** 2022-07-05

**Authors:** Leila M. Reyes Ruiz, Kathleen A. King, Christian Agosto-Burgos, Isabella S. Gamez, Nicole C. Gadda, Elizabeth M. Garrett, Rita Tamayo

**Affiliations:** 1 Department of Microbiology and Immunology, University of North Carolina School of Medicine, Chapel Hill, North Carolina, United States of America; 2 Department of Pathology and Laboratory Medicine, University of North Carolina School of Medicine, Chapel Hill, North Carolina, United States of America; University of Maryland, UNITED STATES

## Abstract

The opportunistic nosocomial pathogen *Clostridioides difficile* exhibits phenotypic heterogeneity through phase variation, a stochastic, reversible process that modulates expression. In *C*. *difficile*, multiple sequences in the genome undergo inversion through site-specific recombination. Two such loci lie upstream of *pdcB* and *pdcC*, which encode phosphodiesterases (PDEs) that degrade the signaling molecule c-di-GMP. Numerous phenotypes are influenced by c-di-GMP in *C*. *difficile* including cell and colony morphology, motility, colonization, and virulence. In this study, we aimed to assess whether PdcB phase varies, identify the mechanism of regulation, and determine the effects on intracellular c-di-GMP levels and regulated phenotypes. We found that expression of *pdcB* is heterogeneous and the orientation of the invertible sequence, or ‘*pdcB* switch’, determines expression. The *pdcB* switch contains a promoter that when properly oriented promotes *pdcB* expression. Expression is augmented by an additional promoter upstream of the *pdcB* switch. Mutation of nucleotides at the site of recombination resulted in phase-locked strains with significant differences in *pdcB* expression. Characterization of these mutants showed that the *pdcB* locked-ON mutant has reduced intracellular c-di-GMP compared to the locked-OFF mutant, consistent with increased and decreased PdcB activity, respectively. These alterations in c-di-GMP had concomitant effects on multiple known c-di-GMP regulated processes, indicating that phase variation of PdcB allows *C*. *difficile* to coordinately diversify multiple phenotypes in the population to enhance survival.

## Author summary

Phase variation is a mechanism by which many bacteria introduce phenotypic heterogeneity into a population as a bet-hedging strategy to help ensure survival under detrimental conditions. In *C*. *difficile*, the intracellular signaling molecule c-di-GMP regulates production of flagella, toxins, adhesins, and other factors that impact virulence. *C*. *difficile* encodes multiple c-di-GMP synthases and hydrolases that modulate intracellular c-di-GMPs and control these processes. Here, we show that production of a c-di-GMP hydrolytic enzyme, PdcB, undergoes phase variation in *C*. *difficile*. We generated phase-locked mutants unable to phase vary and found that PdcB affects global intracellular c-di-GMP levels, swimming and surface motility, and biofilm formation. These findings suggest that phase variation of PdcB enables *C*. *difficile* to coordinately regulate the production multiple factors by generating heterogeneity in intracellular c-di-GMP levels among bacteria in the population.

## Introduction

*Clostridioides difficile* is a gram-positive, spore-forming, obligate anaerobe that causes antibiotic-associated intestinal disease ranging from mild diarrhea to pseudomembranous colitis in susceptible hosts. Several recent studies have shown that *C*. *difficile* exhibits heterogeneity in gene expression and associated phenotypes [1–4]. Phase variation is one mechanism by which bacteria introduce phenotypic heterogeneity into a clonal population, helping ensure the survival of the population if exposed to adverse conditions [5,6]. Typically, phase variation modulates the production of surface factors such as flagella, fimbriae, and exopolysaccharides; in host-associated bacteria, the process can influence immune system evasion, colonization, and virulence [5,7]. Several genetic and epigenetic mechanisms can drive phase variation, including conservative site-specific DNA recombination in which a serine or tyrosine recombinase mediates the reversible inversion of a DNA sequence [8,9]. The invertible sequence, or “switch”, is flanked by short inverted repeats and contains the information for regulating the expression of the adjacent gene or operon.

In the *C*. *difficile* epidemic-associated strain R20291, seven sequences undergo inversion [1,2,10], and three have been shown to mediate phase variation [1,2,4]. These switches modulate expression of: *cwpV*, which encodes a cell wall protein involved in bacteriophage resistance [1,11]; *cmrRST*, which encodes an atypical signal transduction system that impacts cell and colony morphology, surface motility, flagellar motility, and virulence [4,10]; and the *flgB* operon, a large operon encoding flagellar hook and basal body components as well as the sigma factor SigD [2,12,13]. SigD coordinates expression of additional flagellar operons and positively regulates expression of the *tcdA* and *tcdB* toxin genes [14,15]. Accordingly, the production of the glucosylating toxins required for virulence is indirectly subject to phase variation via the flagellar (*flg*) switch [2,12]. Phase variation via the *cwpV* and *flg* switches occurs through RNA-mediated mechanisms post-transcription initiation, while a promoter within the *cmr* switch mediates phase variation of CmrRST [1,2,13,16].

The signaling molecule cyclic diguanylate monophosphate (c-di-GMP) regulates the transition between motile and sessile lifestyles in numerous bacterial species [17,18]. c-di-GMP is synthesized by diguanylate cyclases (DGCs) containing a GGDEF domain and degraded by phosphodiesterases (PDEs) containing an EAL or HD-GYP domain [18]. These enzymes are regulated at the transcriptional and post-translational levels, and their opposing activities control the intracellular c-di-GMP concentration. Like many bacterial species, *C*. *difficile* encodes dozens of c-di-GMP metabolic enzymes—the ribotype 027 strain used in this study, R20291, encodes 15 known or putative DGCs and 17 known or putative PDEs [19,20]. In *C*. *difficile*, regulation of gene expression by c-di-GMP is largely mediated by riboswitches that either increase or decrease gene expression when c-di-GMP is bound [21–24]. The majority of genes directly regulated by a c-di-GMP riboswitch encode surface-associated factors that impact adhesion or motility. Also as in other bacterial species, c-di-GMP has broad regulatory effects in *C*. *difficile*, inhibiting flagellar gene expression, swimming motility, toxin production and sporulation [25–27], and increasing expression of adhesin genes and associated surface behaviors such as biofilm formation [28–33].

Phase variation and c-di-GMP signaling appear to be linked in *C*. *difficile*. Expression of the *flgB* and *cmrRST* operons is regulated by both c-di-GMP and phase variation [23,25,34]. In addition, two of the genes encoding proteins with tandem GGDEF and EAL domains, CDR20291_0685 (*pdcB*) and CDR20291_1514 (*pdcC*), are preceded by invertible elements and are thus likely subject to phase variation [10,35]. Both PdcB and PdcC contain a degenerate GGDEF domain lacking nucleotides required for DGC activity and a conserved EAL domain [19]. Overexpression of the orthologous genes from *C*. *difficile* 630 in both *Vibrio cholerae* and *Bacillus subtilis*, which have well-described responses to c-di-GMP, resulted in phenotypes consistent with PDE function. In addition, PdcB (orthologue CD630_0757) exhibited c-di-GMP hydrolytic but not synthase activity *in vitro* [19,36]. Phase variation of these PDEs is therefore poised to modulate one or more c-di-GMP-regulated phenotypes.

In this study, we aimed to determine whether PdcB is subject to phase variation and the potential consequence on c-di-GMP signaling and *C*. *difficile* physiology and behavior. We found that the upstream invertible element, the “*pdcB* switch”, is found in both orientations in multiple *C*. *difficile* strains. We demonstrated that *pdcB* is expressed heterogeneously in *C*. *difficile*, identified the switch orientation that promotes *pdcB* expression, and determined the mechanism by which the switch regulates expression. By mutating key nucleotides in the right inverted repeat, we generated *pdcB* phase-locked strains that are incapable of switch inversion, and these mutants differed significantly in *pdcB* expression. Using a riboswitch-based fluorescent reporter in single-cell and bulk-population analyses, we found that the phase-locked ON mutant had significantly lower c-di-GMP compared to the phase-locked OFF mutant. Consistent with these differences in c-di-GMP, the phase-locked ON mutant showed reduced swimming motility, but increased surface motility than the phase-locked OFF mutant. Throughout this study, we accounted for the potential contributions of the multiple other phase variable factors, particularly PdcC and others that might influence motility and biofilm phenotypes. Together these results indicate that *C*. *difficile* employs phase variation of a c-di-GMP PDE to coordinately modulate multiple mechanisms of adaptation to environmental stimuli.

## Results

### The orientation of the *pdcB* switch is heterogeneous in multiple *C*. *difficile* strains

**Fig 1:**
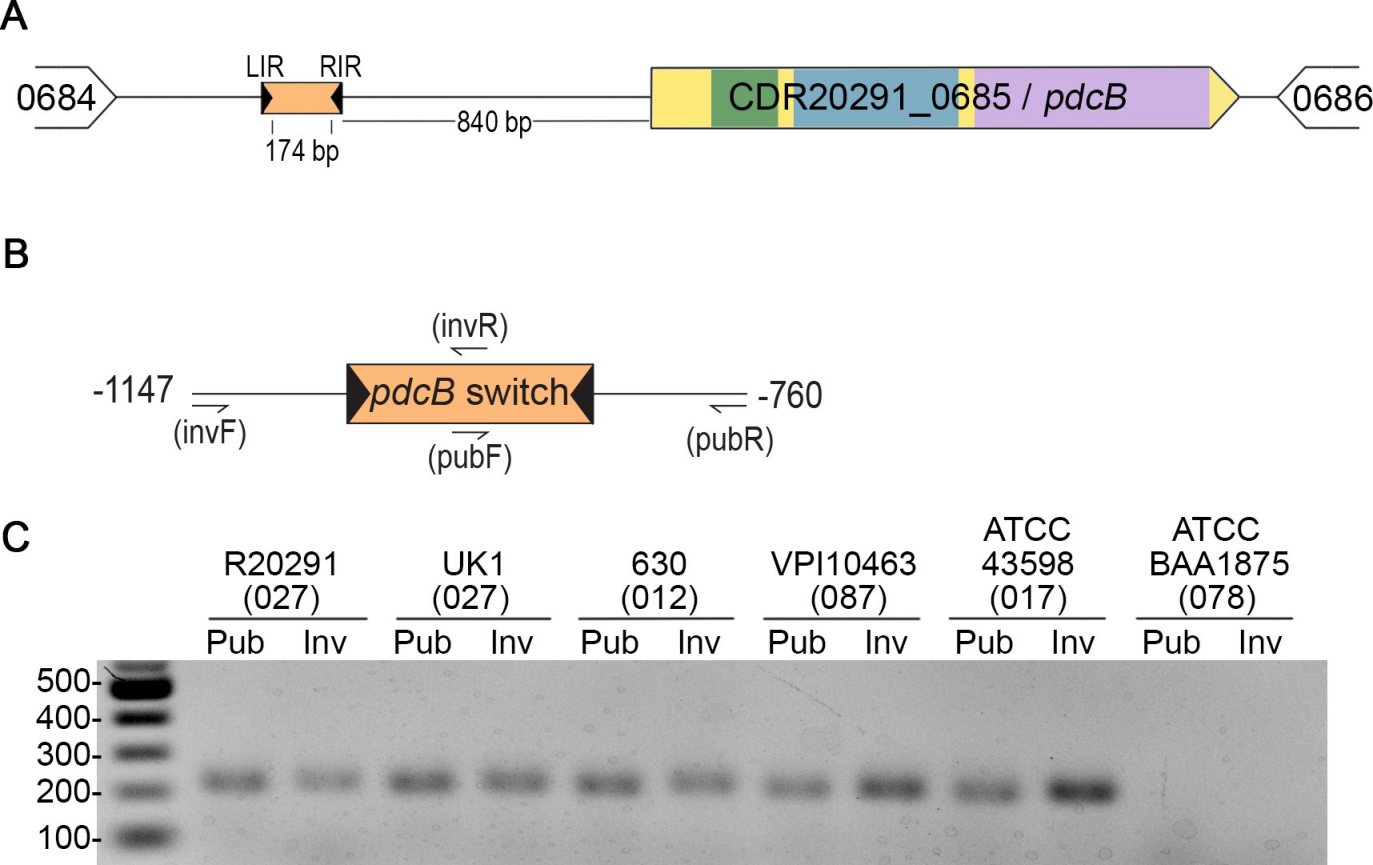
The *pdcB* switch (Cdi2) is invertible in *C*. *difficile*. (A) Diagram of the *pdcB* locus indicating the relative positions of the upstream invertible element (orange) and inverted repeats (black arrows). LIR–left inverted repeat, RIR–right inverted repeat. PdcB contains a PAS domain (green), a degenerate GGDEF domain (blue), and an EAL domain (purple). (B) Diagram of the OS-PCR strategy used in (C) to selectively amplify the orientation of the *pdcB* switch present in the published genome of R20291 (Pub) or the inverse (Inv) orientations. Primer positions are indicated by half-arrows, and the genomic region indicated (-1147 to -760) is relative to the predicted *pdcB* start codon. (C) OS-PCR for the *pdcB* switch in six different *C*. *difficile* strains, with the previously determined ribotype indicated in parenthesis.

The *pdcB* gene encodes a c-di-GMP phosphodiesterase (PDE) containing a PAS sensory domain, a degenerate GGDEF domain, and an EAL domain (Fig 1A) [19]. In *C*. *difficile* R20291, an epidemic-associated ribotype 027 strain, the invertible element Cdi2 is 174 bp long, flanked by imperfect 19 bp inverted repeats, and located 840 bp upstream of the *pdcB* coding sequence (Fig 1A) [10,37]. Sekulovic et al. previously showed that the Cdi2 sequence can be found in both orientations in R20291, suggesting that the sequence undergoes inversion [10]. To determine whether the Cdi2 sequence is similarly heterogeneous in other *C*. *difficile* strains, we employed orientation-specific PCR (OS-PCR), which uses primer sets that allow the differential amplification of either Cdi2 sequence orientation, qualitatively showing the presence or absence of each (Fig 1B) [1,2,38]. We selected four additional *C*. *difficile* strains representing multiple ribotypes (RT): UK1 (RT027), 630 (RT012), VPI10463 (RT087), and ATCC43598 (RT017). ATCC BAA1875 (RT078), which lacks an orthologous *pdcB* locus, was included as a negative control. The Cdi2 sequence was detected in both orientations in all but the BAA1875 negative control (Fig 1C). This heterogeneity in the orientation of the Cdi2 sequence, herein named the “*pdcB* switch”, suggests that the sequence undergoes inversion in multiple *C*. *difficile* strains when grown in standard laboratory conditions.

### *pdcB* switch orientation modulates *pdcB* expression

To quantitatively assess the heterogeneity in *pdcB* switch orientation and its impact on *pdcB* expression, we isolated six independent colonies of R20291 grown on BHIS-agar and collected paired samples for genomic DNA and RNA isolation. The genomic DNA was subjected to quantitative PCR using the orientation-specific primers described above (OS-qPCR) to determine the percentage of a population with the *pdcB* switch in the orientation present in the R20291 reference genome (accession number FN545816). The abundance of *pdcB* transcript in the matched RNA sample was evaluated by quantitative reverse transcriptase PCR (qRT-PCR) and normalized to the abundance of *rpoC* transcript.

**Fig 2:**
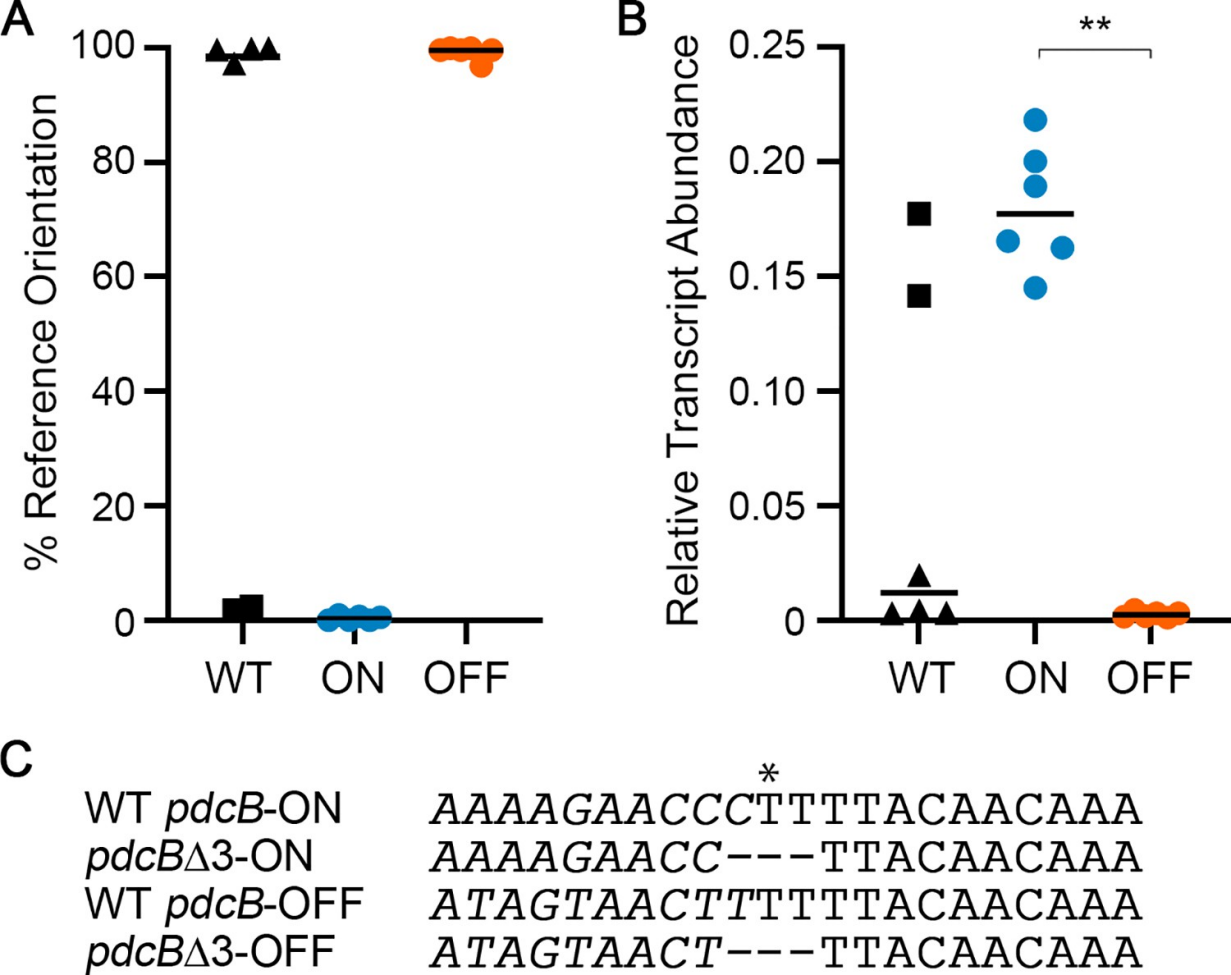
Mutation of the right inverted repeat results in phase-locked strains. **(**A) OS-qPCR was performed using genomic DNA from R20291 (WT), *pdcB*Δ3-ON, and *pdcB*Δ3-OFF. Data are expressed as the percentage of the invertible element in orientation present in the reference genome (Pub/OFF). (B) qRT-PCR was performed using cDNA derived from R20291 (WT), *pdcB*Δ3-ON, and *pdcB*Δ3-OFF. Data are expressed as the ratio of the *pdcB* transcript abundance to that of the *rpoC* reference gene. (C) Three nucleotides in the RIR were deleted to create strains with the invertible element locked in the ON and OFF orientations. Asterisk indicates the site of recombination [10]. Italics indicate a portion of the *pdcB* switch sequence that undergoes inversion. (A, B) Symbols represent values from independent samples. For WT, triangles and squares distinguish isolates with reference and inverse sequence orientations, respectively. Indicated are the medians. **p < 0.01 by Kruskal-Wallis test and Dunn’s post-test.

Of the six isolates, four contained the *pdcB* switch predominantly in the reference genome orientation (99.22% ± 1.27% reference) (Fig 2A, WT triangles). These isolates showed low levels of *pdcB* mRNA, 0.8% of *rpoC* transcript levels (Fig 2B, WT triangles). The remaining two isolates had the *pdcB* switch in the inverse orientation (0.38% ± 0.42% reference) (Fig 2A, WT squares). These two isolates had significantly higher *pdcB* transcript levels, 16% of *rpoC* transcript levels (Fig 2B, WT triangles). These results show that *pdcB* switch orientation and expression is bimodal in wild-type *C*. *difficile*.

Due to the heterogeneity of *pdcB* switch orientation, we created mutants in which the *pdcB* switch was locked in the published or inverted orientation to further examine the effect of the switch orientation in *pdcB* expression. Phase-locked mutants have previously been generated by inactivating the *recV* gene encoding the recombinase required for inversion of the *flg* and *cmr* switches [1,2,4]. However, the site-specific recombinase required for *pdcB* switch inversion has not been identified [10]. Sekulovic et al. identified the nucleotide within the inverted repeats where recombination occurs for each switch [10]. Deleting this nucleotide and a nucleotide on each side was recently demonstrated to prevent site-specific recombination and inversion of the *flg* and *cmr* switches [16,39]. We used this strategy to create strains with the equivalent 3-nucleotide deletion in the right inverted repeat (RIR) of the *pdcB* switch, one in which the switch is in the reference genome orientation, and the other with the switch in the inverse orientation (Fig 2C). Whole genome sequencing confirmed the intended mutations and integrity of the genomes compared to the R20291 parent. We then applied OS-qPCR to confirm that the *pdcB* switch in these strains is locked in the desired orientations. As anticipated, the mutant with the *pdcB* switch in the presumed OFF orientation contained the switch in almost exclusively in the reference genome orientation (99.27% ± 1.21% reference), and we named this mutant *pdcB*Δ3-OFF (Fig 2A). The mutant with the switch in the presumed ON orientation had the inverted sequence (0.38% ± 0.42% reference), and we named this mutant *pdcB*Δ3-ON (Fig 2A). Both *pdcB*Δ3 mutants expressed *pdcB* consistent with their ON and OFF assignments, with levels equivalent to the naturally arising ON and OFF variants of wild-type R20291 (Fig 2B). Together these findings indicate that *pdcB* switch inversion mediates the phase variable expression of *pdcB*, and that the switch orientation in the R20291 reference genome corresponds to the OFF orientation, and the inverse sequence corresponds to the ON state.

### The *pdcB* switch contains an invertible promoter

**Fig 3:**
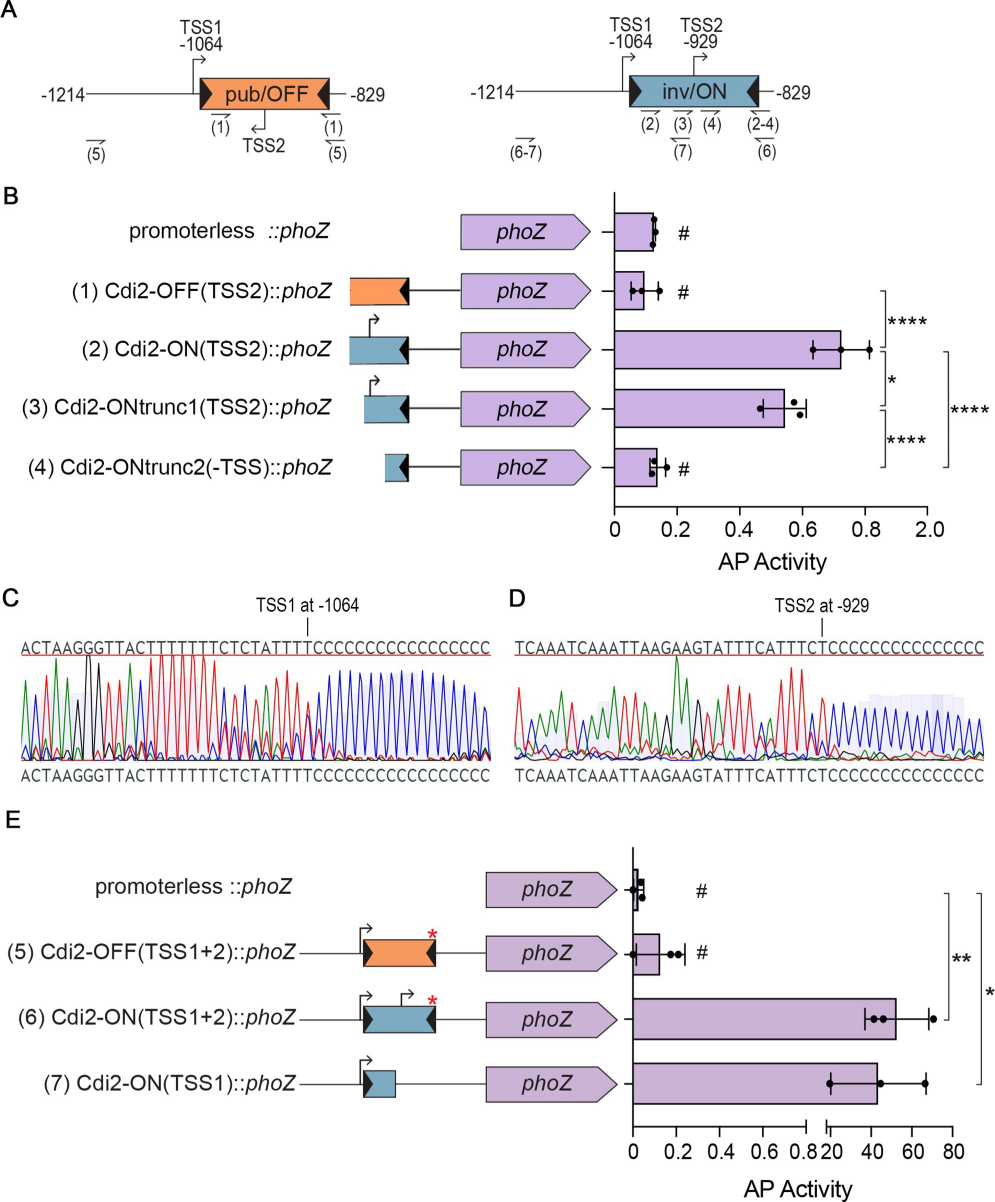
The *pdcB* switch contains an invertible promoter. (A) Diagrams of the *pdcB* switch in the OFF and ON orientations with TSS1 and TSS2 positions indicated relative to the *pdcB* start codon. Positions of primers used to construct the fusions to *phoZ* in (B) and (E) are indicated by half arrows; numbers in parentheses match the primers used with the corresponding plasmid construct. (B) Alkaline phosphatase (AP) assay using *C*. *difficile* strains with plasmid-borne transcriptional fusions to *phoZ*. Promoterless *phoZ* was used as control. (C,D) Chromatographs of the Sanger sequencing results obtained by 5’ RACE from (C) wild-type R20291 and (D) R20291 carrying the Cdi2-ON::*phoZ* reporter. TSS1 and TSS2 were identified as the first nucleotide adjacent to the poly-C tail added to the 5’ end of cDNA; the position relative to the *pdcB* start codon is indicated. (E) AP assay using *C*. *difficile* strains with plasmid-borne transcriptional fusions to *phoZ*. Red asterisks indicate the presence of a 3-nucleotide deletion in the RIR. (B,E) Means and standard deviations from 3 independent experiments are shown. *p < 0.05, **p < 0.01, ****p < 0.0001 by one-way ANOVA and Tukey’s post-test. No significant differences between strains marked #. The nucleotide positions relative to the *pdcB* predicted start codon for each end (5’, 3’) of the *C*. *difficile* sequences fused to *phoZ* are as follows: (1) -1,024 to -829, (2) -1,023 to -829, (3) -974 to -829, (4) -915 to -829, (5) -1,214 to -829, (6) -1,214 to -829, and (7) -1,214 to -957.

In many examples of phase variation by site-specific DNA recombination, the invertible element contains a promoter that, when properly oriented, promotes transcription of the adjacent gene(s) leading to the phase ON state; in the other orientation, the promoter is directed away from the gene(s) resulting in the phase OFF state [5]. To determine if a promoter is present in the *pdcB* switch, we generated plasmid-borne transcriptional reporters in which the *pdcB* switch is fused to the *phoZ* reporter gene encoding an alkaline phosphatase (AP). Fusions were made for the *pdcB* switch in both the OFF orientation (Cdi2-OFF::*phoZ*) and ON orientation (Cdi2-ON::*phoZ*) (Fig 3A and 3B). Pilot experiments with longer constructs indicated that the *pdcB* switch in these reporter constructs readily inverted when introduced into R20291. To prevent switch inversion during the experiments, the left inverted repeat (LIR) was excluded from the constructs. A promoterless fusion was included as a control (promoterless:: *phoZ*). Each of these plasmids was introduced into R20291, and AP activity was measured in the resulting strains. We found that when the *pdcB* switch was in the OFF orientation, AP activity was similar to the promoterless control; when in the ON orientation, AP activity increased 6-fold, indicating the presence of an active promoter in the ON, but not OFF, *pdcB* switch orientation (Fig 3B). To begin to determine the location of the putative promoter in the ON orientation, we made serial truncations from the 5’ end of the *pdcB* switch in the ON orientation (Cdi2-ONtrunc1::*phoZ* and Cdi2-ONtrunc2::*phoZ*). Removal of one-third of the *pdcB* ON switch sequence resulted in a 25% reduction in AP activity compared to the full-length construct, though the level of activity remained significantly higher than the Cdi2-OFF and promoterless reporters (Fig 3B). In contrast, removal of two-thirds of the *pdcB* ON switch sequence reduced AP activity to the levels of the promoterless control. Together these data indicate that a promoter is present near the center of the *pdcB* ON switch.

To precisely map the position of the promoter present in the *pdcB* switch, we used 5’ Rapid Amplification of cDNA Ends (5’ RACE) to identify transcriptional start sites (TSS) in wild-type R20291. Because of the distance of the *pdcB* switch from the *pdcB* coding sequence, we used primers within 300 bp of the RIR. Using this strategy, we detected a TSS at position -1064 upstream of the annotated *pdcB* start codon (Fig 3A and 3C, S1 Fig). This TSS (TSS1) is located upstream of the *pdcB* switch, 26 nucleotides 5’ of the LIR. We manually identified a potential -10 and -35 sequences of a σ70 promoter upstream of TSS1 (S1 Fig). We postulated that the TSS1 promoter might mask the signal from a promoter present in the *pdcB* switch, particularly since only a subpopulation of wild-type R20291 contains the switch in the ON orientation. To identify a TSS located specifically in the invertible element, we performed 5’ RACE using RNA extracted from *C*. *difficile* R20291 strains carrying the Cdi2-OFF::*phoZ* and Cdi2-ON::*phoZ* reporters and a primer that anneals to *phoZ* to identify any TSS from *phoZ* transcripts and not from the native locus. With this modification, we identified a TSS (TSS2) from Cdi2-ON::*phoZ* corresponding to position -929 upstream of the predicted *pdcB* start codon (Figs 3A, 3D and S1). No TSS was identified from Cdi2-OFF::*phoZ*. These results indicate that at least two promoters regulate transcription of *pdcB*, one present when the switch is in the inverted/ON orientation and a second one present upstream of the *pdcB* switch (Fig 3A).

To test the activity of the TSS identified upstream of the *pdcB* switch, TSS1, we generated additional *C*. *difficile* strains with plasmid-borne *phoZ* transcriptional reporters of TSS1 alone or combined with the *pdcB* switch in either the ON or OFF orientation. Because both inverted repeats were included, these fusions contain the 3-nucleotide deletions in the RIR present in *pdcB*Δ3-OFF and *pdcB*Δ3-ON strains to prevent switching of the invertible element. We found that when TSS1 is combined with the *pdcB* OFF switch orientation, AP activity was equivalent to the vector control. In contrast, TSS1 alone or in combination with the *pdcB* ON switch (TSS2) resulted in increased AP activity compared to the OFF orientation or vector control. These data indicate that TSS1 located upstream of the *pdcB* switch is functional and that its activity depends on the orientation of the *pdcB* switch.

### *pdcB* expression affects intracellular c-di-GMP levels

Phase variation of PdcB is poised to modulate intracellular c-di-GMP levels. To test the effect of *pdcB* expression on c-di-GMP levels, we used a c-di-GMP riboswitch-based biosensor that allows measurements at the single cell level and in the bulk population. The biosensor, P_*gluD*_-PRS::mCherryOpt, is encoded on a multi-copy plasmid and consists of three elements: the heterologous *gluD* promoter that is not affected by c-di-GMP [23], the leader sequence of *pilA1* containing the Cdi-2-4 riboswitch that promotes gene expression in response to c-di-GMP [23,28], and the mCherryOpt gene encoding a red fluorescence protein codon-optimized for translation in *C*. *difficile* [40]. An identical reporter with a mutation of a residue required for c-di-GMP binding was included as a negative control (P_*gluD*_-PRS^A70G^::mCherryOpt) [28,29]. The respective pPRS::mCherryOpt and pPRS^A70G^::mCherryOpt constructs were each introduced into WT, *pdcB*Δ3-OFF, and *pdcB*Δ3-ON.

**Fig 4:**
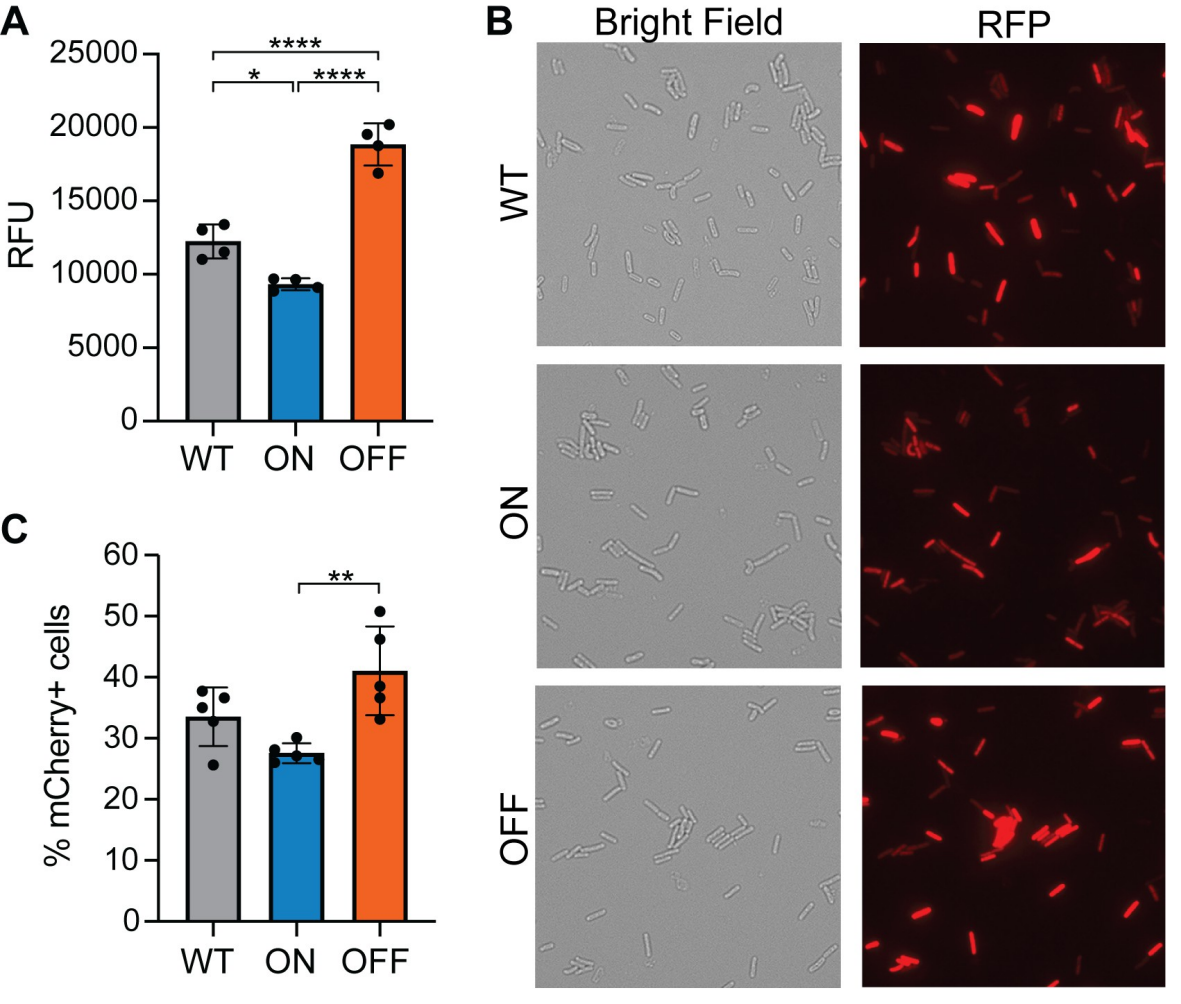
Phase variation of PdcB modulates c-di-GMP at the population and single-cell levels. (A) Measurement of c-di-GMP using the pPRS::mCherry reporter in R20291 (WT), *pdcB*Δ3-ON, and *pdcB*Δ3-OFF. Relative Fluorescence Units (RFU) calculated from arbitrary fluorescence units after 180 minutes RFP maturation normalized to OD_600_ at time 0. Kinetic analysis of fluorescence and data for the pPRS^A70G^::mCherry controls are in S2 Fig. (B) Representative micrographs showing heterogeneity in fluorescence in the above strains. (C) Number of mCherryOpt-expressing cells determined by flow cytometry expressed as a percentage of total Syto-9 stained cells. (A,C) Bars indicate means and standard deviations, with circles indicating values from independent biological samples. *p< 0.05, **p< 0.01, ****p<0.0001 by one-way ANOVA and Tukey’s post-test.

To measure c-di-GMP levels in these strains, we used a previously described assay to quantify red fluorescence normalized to the optical density of the culture [40,41]. For P_*gluD*_-PRS::mCherryOpt in the *pdcB*Δ3-ON background, we detected a 24% reduction in fluorescence compared to WT, consistent with increased production of PdcB and a corresponding reduction in c-di-GMP (Figs 4A and S2). In the *pdcB*Δ3-OFF background, fluorescence was 65% higher than in WT, indicating increased c-di-GMP as a result of decreased PdcB (Figs 4A and S2). The equivalent strains bearing P_*gluD*_-PRS^A70G^::mCherryOpt did not exhibit fluorescence above that of the R20291 control without the mCherryOpt plasmid (S2 Fig). Thus, these reporters provided sensitive and specific detection of c-di-GMP *in vivo*, and these results indicate that phase variation of PdcB significantly alters the global intracellular concentration of c-di-GMP in *C*. *difficile*.

Given the heterogeneity of *pdcB* expression in wild-type R20291, the differences in c-di-GMP-mediated fluorescence may arise from an increase in c-di-GMP in all cells, an increase in c-di-GMP in a subset of bacteria that increases the average of the population, or both. To distinguish between these possibilities, we examined fluorescence of individual cells of WT, *pdcB*Δ3-ON, and *pdcB*Δ3-OFF bearing P_*gluD*_-PRS::mCherryOpt by microscopy. Similar proportions of total bacteria exhibited some fluorescence: WT, 95.3% ± 2.1%; *pdcB*Δ3-ON, 93.4% ± 2.2%; and *pdcB*Δ3-OFF, 93.9% ± 0.2%) (Fig 4B). However, we observed heterogeneity in the intensity of mCherry fluorescence across cells in the population (Fig 4B). To quantify this heterogeneity and account for potential differences fluorophore maturation time, we performed flow cytometry to determine the number of cells with mCherryOpt expression. The percentage of fluorescent bacteria in the population is consistent with the expected changes in c-di-GMP with *pdcB*Δ3-OFF samples showing a significantly higher percentage of mCherryOpt-positive bacteria (41.0 ± 7.3%) compared to the *pdcB*Δ3-ON samples (27.6 ± 1.6%) (Figs 4C and S3). The WT showed intermediate fluorescent bacteria. Furthermore, the median fluorescence intensity was 14.2% higher in the *pdcB* OFF population than in the *pdcB* ON population (p < 0.0001) and 10.3% higher than in WT (p < 0.001).

### Phase variation of PdcB modulates multiple c-di-GMP regulated processes

Because the *pdcB*Δ3 mutations significantly altered global c-di-GMP levels, we evaluated the effects of PdcB phase variation on representative c-di-GMP regulated phenotypes: swimming motility and toxin production which are negatively regulated by c-di-GMP, and surface motility and biofilm formation which are positively regulated by c-di-GMP [25,29,34]. Because *pdcB* expression can occur independently of the *pdcB* switch through the upstream promoter (Fig 3), we included a Δ*pdcB* control in these assays.

**Fig 5:**
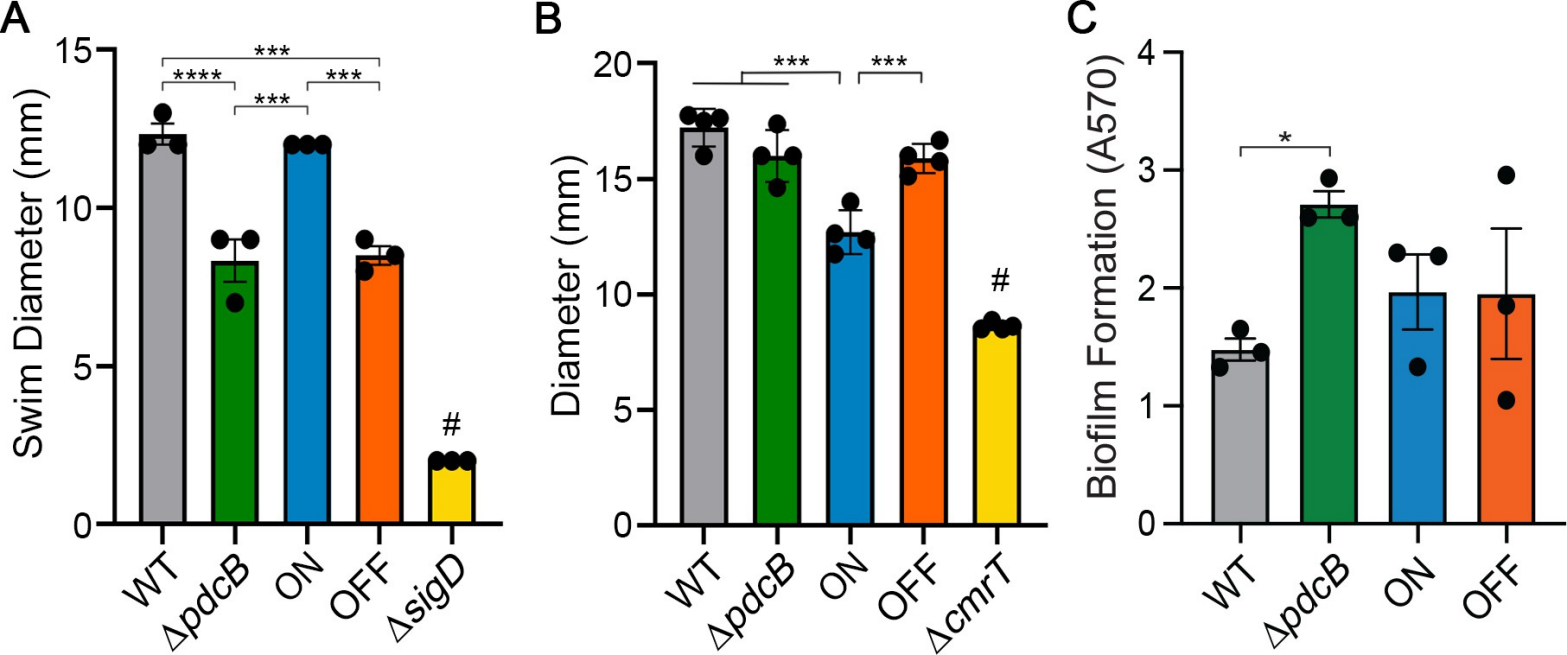
Expression of *pdcB* affects known c-di-GMP regulated processes. Phenotypic analysis of *C*. *difficile* R20291 (WT), Δ*pdcB*, *pdcB*Δ3-ON (ON), and *pdcB*Δ3-OFF (OFF). (A) Swimming motility in 0.5x BHIS-0.3% agar after 48 hours. A non-motile mutant (Δ*sigD*) served as a negative control. (B) Surface motility on BHIS-1.8% agar-1% glucose after 6 days. A Δ*cmrT* mutant served as a negative control. (C) Biofilm formation measured by crystal violet staining after 24 hours of growth in BHIS broth. (A-C) Means and error are shown. Symbols indicate values from independent biological samples. *p < 0.05, ***p < 0.001, ****p < 0.0001 by one-way ANOVA and Tukey’s post-test; #*p* < 0.05 compared to all other strains.

The Δ*pdcB* and *pdcB*Δ3-OFF mutants showed comparable swimming motility in BHIS-0.3% agar medium, and both were significantly less motile than WT and *pdcB*Δ3-ON which exhibited similar motility to each other (Fig 5A). Because of the link between flagellum and toxin gene expression, we measured toxin titers in the supernatants of these strains using a Vero cell rounding assay. Toxin titers were significantly lower in Δ*pdcB* supernatants compared to WT (S4 Fig). However, *pdcB*Δ3-ON and *pdcB*Δ3-OFF showed comparable levels of toxin production, and neither differed significantly from WT (S4 Fig).

On a BHIS-1.8% agar surface, Δ*pdcB* and *pdcB*Δ3-OFF exhibited comparable migration, and both showed significantly greater migration than *pdcB*Δ3-ON (Fig 5B). In contrast, WT, *pdcB*Δ3-ON, and *pdcB*Δ3-OFF produced equivalent biofilm biomass (Fig 5C), and Δ*pdcB* showed a significant increase in biofilm compared to WT. These data suggest that PdcB hydrolysis of c-di-GMP promotes swimming motility and inhibits surface motility and biofilm formation, and phase variation of PdcB impacts swimming and surface motility but not biofilm development. Notably, WT matched the swimming phenotype of *pdcB*Δ3-ON but the surface motility phenotype of *pdcB*Δ3-OFF, which may be attributable to the ability of WT to phase vary PdcB.

### Contribution of other phase variable factors

**Fig 6:**
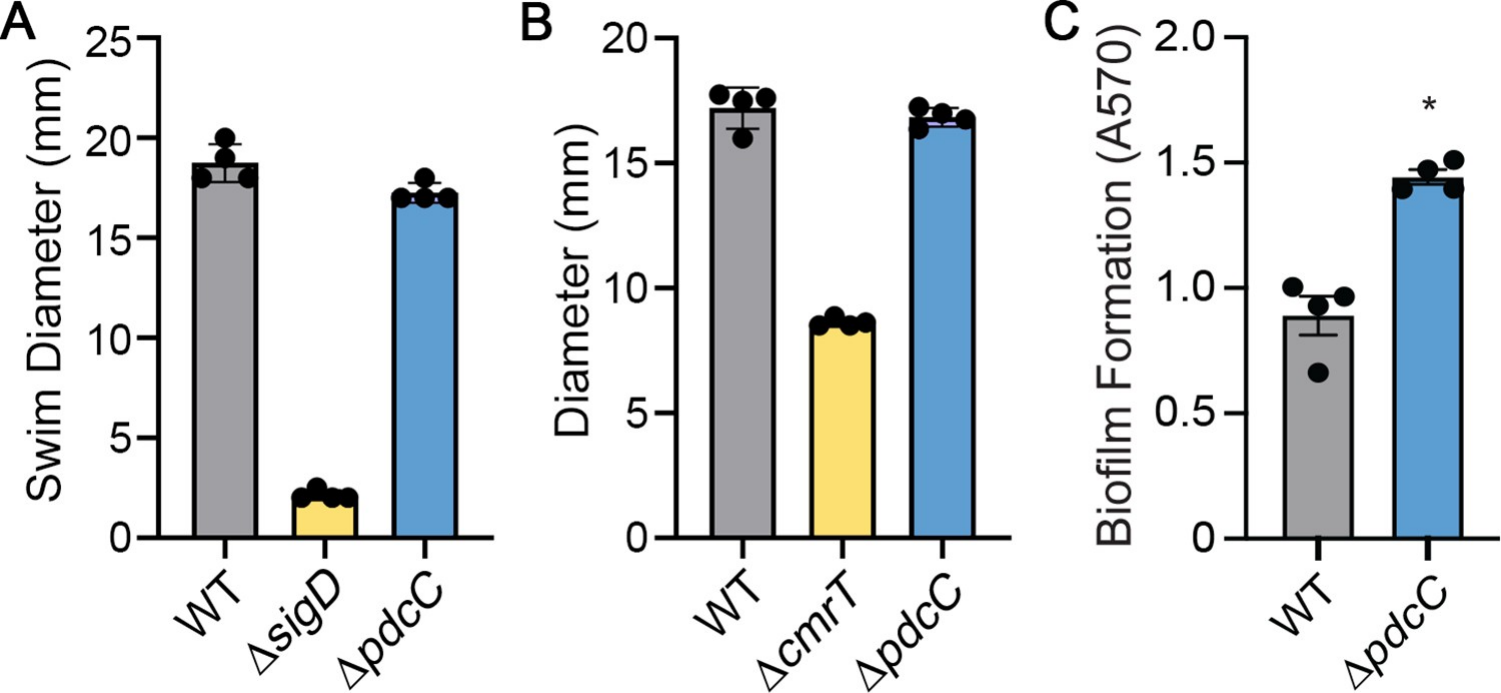
PdcC contributes to inhibition of biofilm formation, but not swimming or surface motility. (A) *S*wimming motility in 0.5x BHIS-0.3% agar after 48 hrs. A non-motile mutant (Δ*sigD*) served as a negative control. (B) Surface motility on BHIS-1.8% agar-1% glucose after 6 days. A Δ*cmrT* mutant served as a negative control. (C) Biofilm formation was measured by crystal violet staining after 24 hours of growth in BHIS broth. (A-C) Means and standard error are shown. Symbols indicate values from independent biological samples. *p < 0.05 by Mann-Whitney test.

In this study we used mutants with fixed orientations of the *pdcB* switch to evaluate the role of PdcB in multiple c-di-GMP regulated processes. However, other phase variable factors could also influence these processes. The *flg* and *cmr* switches have been shown to impact swimming motility, surface migration, and biofilm formation [2,4]. In addition, a second c-di-GMP PDE gene, *pdcC*, is preceded by an invertible sequence [10]. These factors might have phase varied during strain construction, growth in assay conditions, or both, leading to potential misattribution of function to PdcB. The functions of the remaining CDR20291_0963 and CDR20291_3417 loci are not yet known, and the upstream invertible sequences have not been shown to affect expression, so we cannot exclude a role in motility and biofilm formation. To address this caveat, we first addressed the possible contribution of PdcC phase variation. Using *phoZ* reporter assays, we found that orientation of the invertible sequence upstream of *pdcC* modulates gene expression (S5A Fig). In addition, 5’ RACE detected a TSS when the *pdcC* switch is in the orientation that showed transcriptional activity but not the opposite orientation (S5B Fig). Because *pdcC* exhibited phase variable expression, we determined the impact of PdcC on c-di-GMP regulated phenotypes. Deletion of *pdcC* in R20291 had no effect on swimming motility or surface migration (Fig 6A and 6B). However, a Δ*pdcC* mutation led to a 2-fold increase in biofilm formation compared to WT, which is similar to the effect of the *pdcB* mutation on this phenotype (Fig 6C). Therefore, the increased biofilm of the Δ*pdcB* and *pdcB*Δ3-OFF mutants could also be attributed to PdcC.

**Fig 7:**
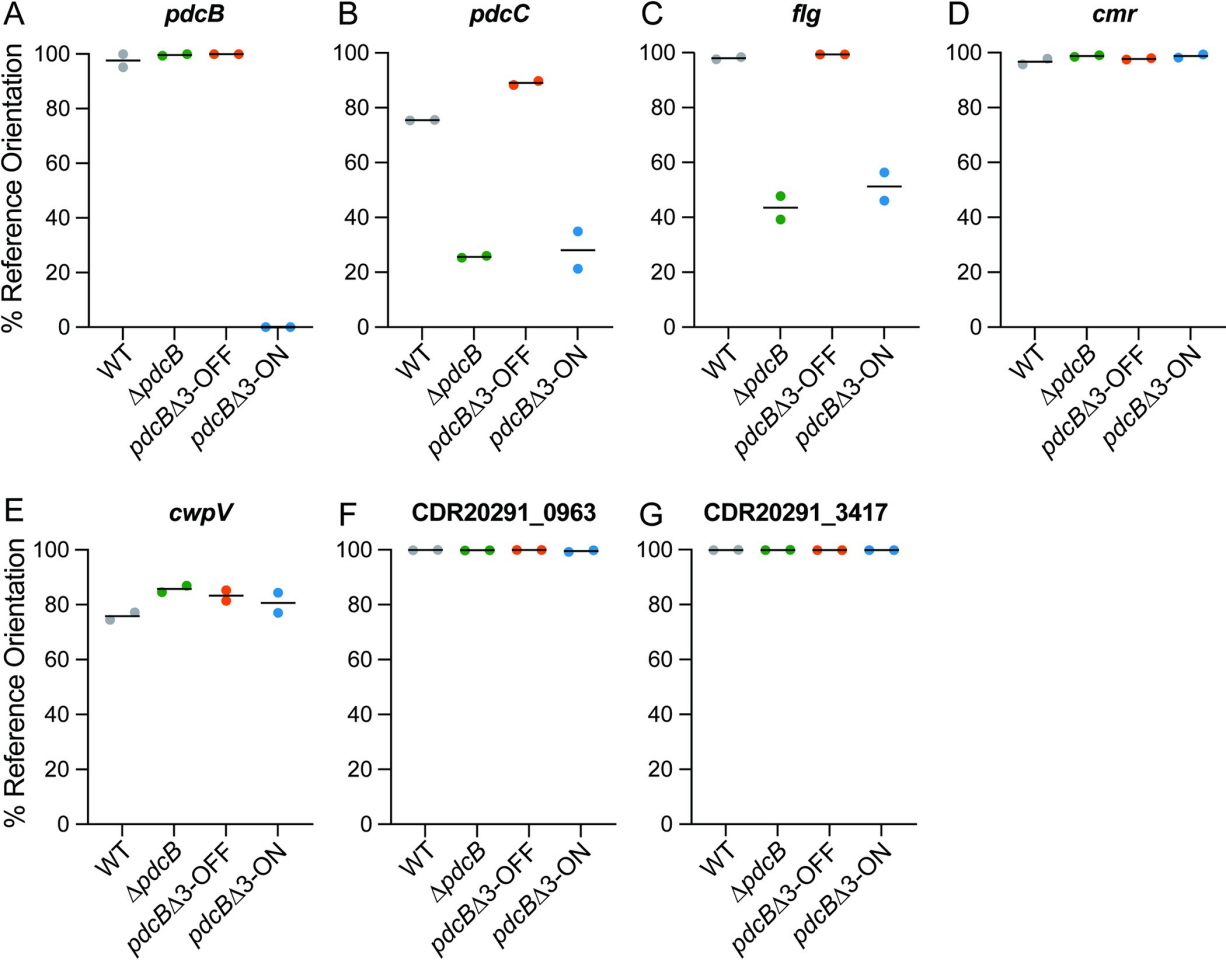
Orientations of the other invertible sequences do not correlate with the phenotypes of the *pdcB* mutants. Genomic DNA from WT, Δ*pdcB*, *pdcB*Δ3-ON, and *pdcB*Δ3-OFF grown on BHIS agar was subjected to OS-qPCR for the invertible elements *pdcB* (A), *pdcC* (B), *flg* (C), *cmr* (D), *cwpV* (E), CDR20291_0963 (F), and CDR20291_3417 (G). The data are presented as the percentage of the invertible element in the orientation of the reference R20291 genome.

We next used OS-qPCR to determine the proportions of the orientations of the *pdcB*, *pdcC*, *flg*, *cmr*, *cwpV*, CDR20291_0963, and CDR20291_3417 invertible sequences present in starting populations of WT, Δ*pdcB*, *pdcB*Δ3-ON, and *pdcB*Δ3-OFF. The *pdcB* switch was almost exclusively in the reference genome (OFF) orientation in all but the *pdcB*Δ3-ON as expected (Fig 7A). In all four strains, the *cmr*, *cwpV*, CDR20291_0963, and CDR20291_3417 sequences were in consistent orientations (Fig 7D–7G), indicating that phenotypic changes observed in the *pdcB* mutants are not a result of phase variation of these sites. The *pdcC* and *flg* switches did differ among the strains, with WT and *pdcB*Δ3-OFF bearing these switches primarily in the reference orientation and Δ*pdcB* and *pdcB*Δ3-ON containing the inverse orientations (Fig 7B and 7C). These results indicate that inversion of the *flg* and *pdcC* switches occurred during the construction of Δ*pdcB* and *pdcB*Δ3-ON. However, the Δ*pdcB* and *pdcB*Δ3-OFF mutants exhibited the same swimming motility and surface motility phenotypes despite having opposite orientations of the flagellar and *pdcC* switches, so these factors are unlikely to have contributed. We cannot dismiss the possibility that phase variation occurred due to selective pressures present during growth in the assay conditions, but assessing the orientations of seven invertible sequences in multiple conditions with sufficient replicates for statistical analysis is imprudent using existing methods.

## Discussion

In this study, we demonstrated that the *pdcB* switch controls phase variation of PdcB, modulating intracellular c-di-GMP levels and multiple known c-di-GMP regulated phenotypes in *C*. *difficile*. The effects of PdcB phase variation on c-di-GMP were apparent at the single cell and population levels. These findings indicate that phase variation of PdcB allows *C*. *difficile* to coordinate the control of multiple factors that play critical roles in motility, surface interactions, and biofilm formation, while diversifying these phenotypes in a population to ensure survival of environmental stresses.

Multiple *C*. *difficile* strains from different ribotypes showed heterogeneity in the orientation of the *pdcB* switch under standard laboratory conditions, suggesting that phase variation of PdcB is conserved and thus important for *C*. *difficile* fitness. However, the BAA1875 strain lacks the *pdcB* locus including the *pdcB* switch. Interestingly, this 078 ribotype strain also is non-motile due to loss of flagellar genes. Given that flagellar genes and swimming motility are inhibited by c-di-GMP, BAA1875 may not experience the same selective pressures as strains that encode flagella. As a result, BAA1875, and perhaps other strains lacking flagella, may not need to modulate c-di-GMP to the same extent.

Analysis of paired DNA and RNA samples from single colonies showed that the ribotype 027 strain R20291 exhibits considerable heterogeneity in *pdcB* switch orientation and *pdcB* expression. Colonies with the *pdcB* switch in the reference genome orientation had lower *pdcB* transcript levels than colonies with the *pdcB* switch in inverse orientation, defining the OFF and ON states of the *pdcB* switch, respectively. These ON and OFF assignments are in agreement with the identification of a transcriptional start site (TSS2) within the *pdcB* switch when in the inverse/ON orientation but not in the published/OFF orientation. Further, promoter activity was detected using transcriptional reporters to the inverse/ON *pdcB* switch sequence but not with a reporter to the published/OFF sequence. We identified an additional transcriptional start site (TSS1) upstream of the *pdcB* switch. The activity of this promoter is substantially higher when the *pdcB* switch is in the ON orientation than in the OFF orientation. The TSS1 promoter also showed higher activity than the TSS2 promoter under standard growth conditions in rich medium. This finding raises the possibility that the TSS2 promoter requires transcriptional activation under specific unknown conditions to initiate *pdcB* expression, which is supported by the lack of a σ70 binding site 5’ of TSS2. It is also possible that the TSS2 promoter does not play a major role in phase variable expression of *pdcB*. Instead, the *pdcB* OFF sequence may contain a bindings site(s) for a transcriptional inhibitor that antagonizes transcription from the TSS1 promoter. Recent work identified a CodY binding site within the *pdcB* switch, which the authors propose could inhibit *pdcB* transcription if in the *pdcB*-OFF state [42]. It is also possible that antisense RNA is produced that inhibits transcription when TSS1 and TSS2 are oriented toward each other in close proximity. Whether and how the TSS1 and TSS2 promoters interact to control *pdcB* expression in different environmental conditions is an area of future study.

The region between the *pdcB* switch RIR and the annotated *pdcB* start codon is unusually long: 840 bp. Fuchs et al. performed a genome-wide study of TSS in *C*. *difficile* 630, but no TSS was identified between the *pdcB* predicted start codon and the upstream gene including those identified in this study [43]. No open reading frame or regulatory RNA is predicted in this region, and we did not detect a transcriptional start site between *pdcB* and the RIR using 5’ RACE. It remains possible that this intervening sequence contains a promoter that is not active in the conditions tested, or that the long leader sequence of the mRNA forms a structure capable of further modulating expression.

**Fig 8:**
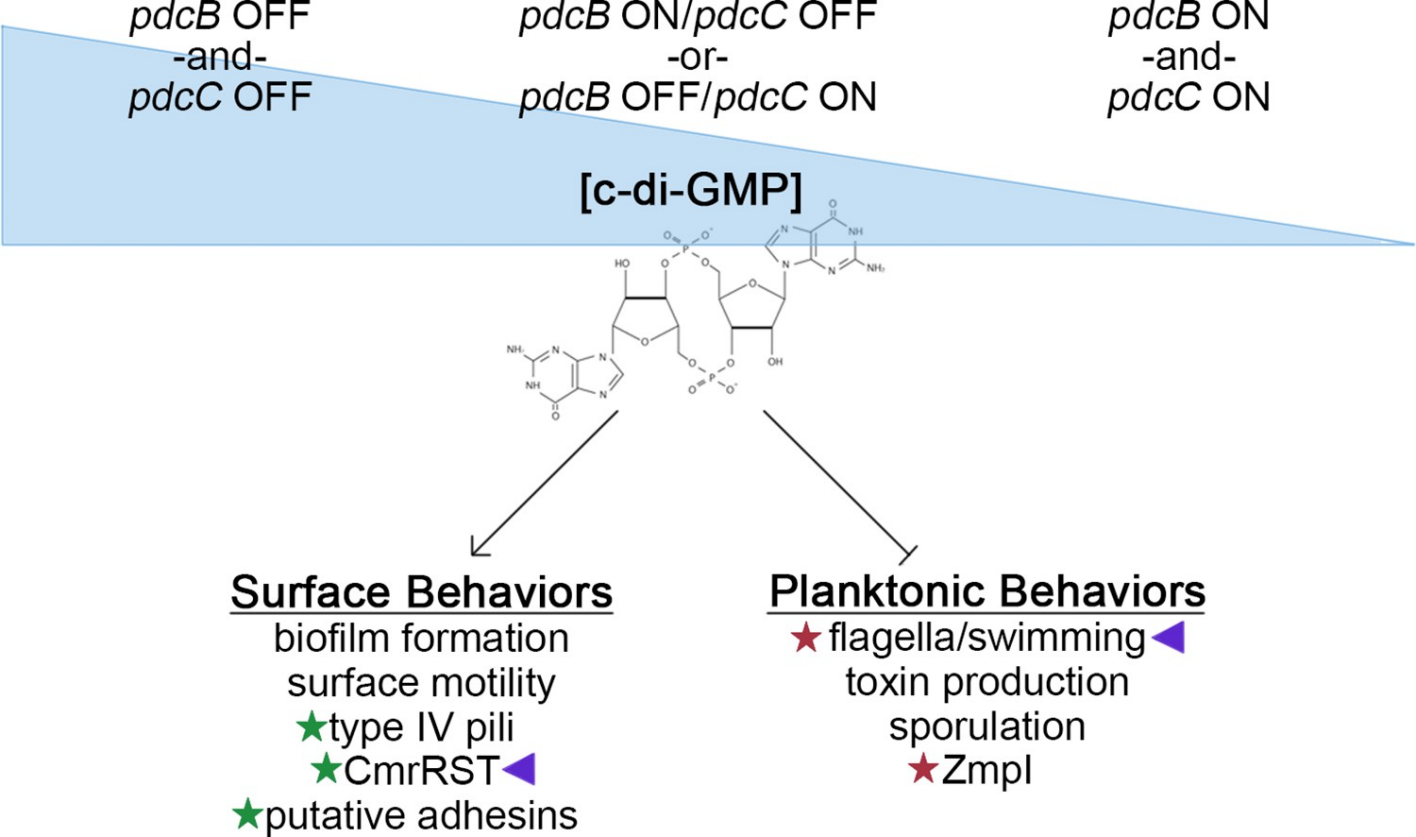
Model of PdcB and PdcC phase variation in the context of c-di-GMP signaling in *C*. *difficile*. Within the range of possible c-di-GMP concentrations established by c-di-GMP biosynthetic and hydrolytic enzymes, represented by the blue wedge, phase variation of PdcB and PdcC may shift c-di-GMP levels in a bacterial cell. A combination of *pdcB* ON and *pdcC* OFF or vice versa would maintain intermediate levels; switching of both to OFF would lead to accumulation of c-di-GMP; and switching of both to ON would deplete c-di-GMP. These switch combinations result in heterogeneity in intracellular c-di-GMP levels in the bacterial population and influence the phenotypes exhibited. Higher c-di-GMP concentrations favor surface-associated behaviors such as biofilm formation and surface migration which are in part mediated by type IV pili, CmrRST, and potentially putative adhesins. Lower c-di-GMP favors planktonic behaviors such as swimming motility, toxin production, sporulation, and the ZmpI metalloprotease proposed to cleave adhesins to release *C*. *difficile* from a surface/biofilm [31,34,50]. Not shown, additional DGCs and c-di-GMP PDEs influence c-di-GMP levels. Green stars indicate factors whose transcription is directly up-regulated by class II c-di-GMP riboswitches; red stars, factors whose transcription is directly down-regulated by class I c-di-GMP riboswitches. Purple triangles denote factors that independently phase vary.

The overall heterogeneity in *pdcB* switch orientation impeded the ability to determine the phenotypic consequences of PdcB phase variation in wild-type R20291 populations. Generating phase-locked strains by deleting 3 nucleotides from the RIR, rendering the *pdcB* switch unable to invert, circumvented this challenge. The phase-locked mutants differed significantly in *pdcB* expression. Using a c-di-GMP riboswitch-based fluorescent reporter, we found that the orientation of the *pdcB* switch modulated the c-di-GMP level in the bulk population consistent with altered PdcB production and c-di-GMP hydrolysis; c-di-GMP was elevated in phase-locked OFF populations with lower *pdcB* expression and reduced in phase-locked ON populations overexpressing *pdcB*. Single-cell analysis using this reporter revealed that the changes in c-di-GMP level were not uniform across the population. Instead, PdcB phase variation resulted in significantly more fluorescent cells in *pdcB*Δ3-OFF (higher c-di-GMP) than in *pdcB*Δ3-ON (lower c-di-GMP) populations. The intracellular c-di-GMP concentration in *C*. *difficile* (strain 630) and ligand affinity of c-di-GMP riboswitches is in the low nanomolar range [21,22,25,44]. The observed differences reflect the proportion of bacteria with c-di-GMP levels above the threshold needed for detection by the *pilA1* c-di-GMP riboswitch, which suggests that PdcB (and PdcC) phase variation affects the proportion of *C*. *difficile* with c-di-GMP above or below a biologically meaningful threshold in the context c-di-GMP heterogeneity generated by numerous c-di-GMP biosynthetic and hydrolytic enzymes (Fig 8).

Phase variation of PdcB affected swimming and surface motility consistent with altered c-di-GMP–the locked-OFF strain, with elevated c-di-GMP, exhibited reduced swimming motility and increased surface motility; the locked-ON mutant showed the opposite phenotypic changes. Flagellum and toxin gene expression are linked, and Δ*pdcB* produced lower toxin titers than WT as expected. However, toxin titers were equivalent in supernatants of WT, *pdcB*Δ3-ON and *pdcB*Δ3-OFF. This discrepancy may be attributed to differences in orientation of the *flg* switch among these strains; swimming motility assay conditions (soft agar medium) present a selective pressure for the *flg*-ON state [2,12], which would restore the *flg* switch to ON in Δ*pdcB* and *pdcB*Δ3-ON and reveal potential effects of PdcB. This selective pressure is absent in broth culture conditions used to measure toxin accumulation (2,12), in which case the *flg*-OFF state could mask any effects of PdcB. Similarly, the *pdcB*-null mutant produced significantly more biofilm than WT, but *pdcB*Δ3-ON and *pdcB*Δ3-OFF produced comparable biofilm. Because the *pdcB* and *pdcC* mutants showed the same increased biofilm phenotype and based on the analysis of *pdcB* and *pdcC* switch orientation, we cannot exclude a role for PdcC. Nonetheless, PdcB and PdcC appear to have distinct regulatory functions as they influence different c-di-GMP regulated phenotypes. Another PDE characterized in *C*. *difficile*, PdcA, also only altered a subset of c-di-GMP regulated phenotypes [45]. The broad (though not universal) regulatory impacts of PdcB suggest that this single PDE allows coordinated phase variation of multiple factors that are known or predicted to contribute to *C*. *difficile* virulence.

Our study highlights a challenge of studying phase variation in *C*. *difficile* in addressing the potential contributions of multiple switches to gene expression and physiology. The *flg* and *cmr* switches impact swimming motility through distinct mechanisms, and PdcB can further regulate flagellar gene expression. The orientation of the *pdcC* switch in the mutants we tested suggests that the swimming and surface motility phenotypes we observed for the *pdcB* mutants were not attributable to *pdcC* expression, though it remains possible that phase variation occurred during growth in the assay conditions. While preparing this manuscript, another report described motility, biofilm, and sporulation phenotypes of a *pdcB* mutant in another 027 ribotype strain, UK1 [42]. Their results were consistent with our findings using the Δ*pdcB* and *pdcB*Δ3-OFF strains in swimming motility assays. However, that work did not demonstrate heterogeneity in *pdcB* expression, nor did it account for phase variation of PdcC or potential inversion of other switches. The colony morphology phenotype they described, for example, may have been attributable to phase variation of CmrRST [4]. Further, our work suggests that differences in biofilm are due to the activity of PdcC and not PdcB. Heterogeneity in switch orientation may be masked by whole genome sequencing without specific efforts to detect inversions [10]. In our study, we took steps to determine the orientations of all seven switches in the strains studied herein. While we cannot exclude the possibility that phase variation occurred during experimentation, we ensured that the starting switch orientations in each strain did not explain the observed phenotypes. Future work will examine the potential selective pressures imposed in these growth conditions on phase variation in *C*. *difficile* [46].

## Materials and methods

### Bacterial growth conditions

*C*. *difficile* strains were grown statically at 37°C in an anaerobic chamber with an anaerobic gas mix of 85% N_2_, 5% CO_2_, and 10% H_2_. Unless otherwise indicated, overnight cultures were grown in Tryptone Yeast (TY) broth, and cultures for experimentation were grown in Brain Heart Infusion medium (Becton Dickinson) supplemented with 0.5% yeast extract (Becton Dickinson) (BHIS). *Escherichia coli* strains were grown in Lucia Bertani (LB) medium (Fisher Scientific) with aeration at 37°C. Antibiotics were used at the following concentrations where indicated: chloramphenicol (Cm) 20 μg/mL, thiamphenicol (Tm) 10 μg/mL, kanamycin (Kan) 100 μg/mL, and ampicillin (Amp) 100 μg/mL. Strains and plasmids used in this study are described in S1 Table.

### DNA manipulation and strain construction

Primers used in plasmid and strain construction were designed based on the *C*. *difficile* R20291 reference genome (Accession No. FN545816) and are listed in S2 Table. To create the plasmids with *pdcB* (Cdi2) and *pdcC* (Cdi3) switch fusions to *phoZ*, the switch regions were PCR amplified from *C*. *difficile* R20291 genomic DNA. The PCR products were cloned into pMC123::*phoZ* (pRT1343) [16] and transformed into DH5α. Transformants were selected on LB-Cm agar and desired clones were identified by PCR and confirmed by sequencing. The plasmids were conjugated into *C*. *difficile* R20291 using *E*. *coli* HB101(pRK24) as the donor [47]. Transconjugants were selected on BHIS-Kan-Tm agar [25]; clones were screen by PCR with primers R837 and R839 to confirm the presence of the plasmid.

To create markerless deletions in the *C*. *difficile* genome, we used the toxin-antitoxin allele replacement system previously described [48]. For deletion of *pdcB* (CDR20291_0685) and *pdcC* (CDR20291_1514), regions upstream and downstream of the locus to be deleted were amplified by PCR using primers named according to the convention locusF1/locusR1 for the upstream region and locus F2/locusR2 for the downstream region (S2 Table). To mutate the RIR of *pdcB* switch, the upstream and downstream regions were amplified with primers introducing the desired mutation. Complementary overlapping sequences were added to the 5’ end of locusR1 and locusF2 primers to allow the products to be spliced together. To lock the *pdcB* switch in the ON orientation, primer sets R3082/R3092 and R3093/R3089 were used to amplify the upstream and downstream fragments, respectively. To lock the *pdcB* switch in the OFF orientation, R3082/R3090 and R3091/R3089 were used. The resulting upstream and downstream fragments were cloned into digested pMSR0 (*BamH*I and *Sac*I) using Gibson Assembly Master Mix (New England BioLabs). The desired clones were confirmed by PCR and sequencing of the inserts. These plasmids were conjugated into *C*. *difficile* strains using *E*. *coli* HB101(pRK24) as the donor. Allelic exchange was done as described [48]. Candidate colonies were screened for the mutant allele using primers R3096/R3097 for Δ*pdcB*, R3146/R3147 for Δ*pdcC*, and R3096/R3138 for *pdcB*Δ3-ON and *pdcB*Δ3-OFF. The integrity of the Δ*pdcB*, Δ*pdcC*, *pdcB*Δ3-ON, and *pdcB*Δ3-OFF genomes were confirmed whole genome sequencing (Microbial Genome Sequencing Center, Pittsburgh, PA).

For the c-di-GMP reporter plasmid pP_*gluD*_-PRS::mCherryOpt and the pP_*gluD*_-PRS^A70G^::mCherryOpt negative control, the P_*gluD*_-PRS and P_*gluD*_-PRS^A70G^ fusions were amplified from previously generated *gusA* reporters containing these sequences, pRT942 and pRT943, respectively, using primers R3063 and R3064. The products were cloned by Gibson assembly into pDSW1728 digested with *Nhe*I and *Sac*I, replacing the existing P*tet* promoter with P_*gluD*_-PRS or P_*gluD*_-PRS^A70G^ [40]. Clones were identified by PCR and confirmed by sequencing of the inserts. The resulting pP_*gluD*_-PRS::mCherryOpt and pP_*gluD*_-PRS^A70G^::mCherryOpt fusions were introduced into R20291, *pdcB*Δ3-ON, and *pdcB*Δ3-OFF by conjugation with *E*. *coli* HB101(pRK24) as described previously, selecting on BHIS-Kan-Tm agar and confirming transconjugants by PCR [25,47].

### Alkaline phosphatase assay

*C*. *difficile* colonies were inoculated into TY broth with Tm (TY-Tm) and grown overnight at 37°C. Cultures were diluted 1:30 into BHIS broth supplemented with Tm (BHIS-Tm) and grown to OD_600_ of approximately 1. Cultures were collected by centrifugation at 16,000 x g for 5 min. The supernatant was discarded and the pellets were stored at -80°C for 24 hrs. Pellets were thawed and the alkaline phosphatase (AP) assay was performed as previously described [49].

### 5’ RACE

Overnight cultures were diluted 1:30 in BHIS broth, with Tm as needed, and grown until OD_600_ of approximately 1. Cells were collected by centrifugation and stored in 1:1 ethanol:acetone. RNA was extracted and purified using the RNeasy Mini Kit (Qiagen) and treated with Turbo DNA-free Kit (Life Technologies). The 5’ RACE System for Rapid Amplification of cDNA Ends kit (Invitrogen) was used to identify transcriptional start sites following the manufacturer’s protocol. Briefly, RNA was denatured, and cDNA was synthesized by reverse transcription using the corresponding primer GSP1. RNA was removed with the provided RNase mix and cDNA was purified using a S.N.A.P column (Invitrogen). A homopolymeric tail was added to the 3’ end of the cDNA using terminal deoxynucleotidyl transferase TdT and deoxycytidine triphosphate (dCTP). Amplification of the cDNA was performed with a primer specific to the oligodC tail (provided by the 5’ RACE System) and a nested gene-specific primer (GSP2 primer corresponding to the TSS identified) (S2 Table). PCR products were purified and sequenced to identify the TSS.

### Determination of switch orientation

Primers used are listed in S2 Table. PCR with orientation-specific primers (OS-PCR) (Fig 1) was done using cell lysates as template [2,10]. Samples of overnight cultures were heated at 98°C for 10 minutes, then 1 μL was used as DNA template for PCR. Thermocycler conditions used were as follows: 98°C for 2 minutes, followed by 30 cycles of 98°C for 30 seconds, 54°C for 15 seconds, and 72°C for 30 seconds.

Quantitative OS-PCR (OS-qPCR) was performed as previously described [10]. Standard OS-qPCR (Fig 2) used 100 ng genomic DNA as the template. When OS-qPCR was performed for all the switches (Fig 7), R20291, Δ*pdcB*, *pdcB*Δ3-OFF, and *pdcB*Δ3-ON were cultured on BHIS agar plates; populations were obtained by collecting 100–150 colonies in 1 mL of BHIS broth. Genomic DNA was purified by phenol:chloroform:isopropanol (25,24,1) extraction and ethanol precipitation. Reactions were set in a total volume of 20 μL that contained 100 ng DNA and primers at a final concentration of 100 nM each (S2 Table). Reactions were run in a Lightcycler 96 system (Roche) with the following conditions: 98°C for 2 minutes followed by 40 cycles of 98°C for 30 seconds, 60°C for 60 seconds and 72°C for 30 seconds. A melting curve was performed as follows: 95°C for 10 seconds, 65°C for 60 seconds and 97°C for 1 second. The *rpoA* gene served as the reference. Data are expressed as the percentage of the indicated switch in the orientation present in the R20291 reference genome [10].

### qRT-PCR

Overnight cultures grown in TY broth were diluted 1:30 in BHIS broth and grown to an OD_600_ of ~ 1. Cells were collected by centrifugation, suspended in 1:1 ethanol-acetone, and stored at -80°C. RNA extraction, DNase treatment, and reverse transcription was performed as previously described [47]. Real-time PCR reactions contained 4 ng of cDNA, primers at a final concentration of 500 nM each, and SYBR Green Real-Time qPCR reagents (Thermo Fisher). Reactions were run in a Lightcycler 96 system (Roche) as follows: 95°C for 10 minutes followed by 40 cycles of 95°C for 30 seconds, 55°C for 60 seconds and 72°C for 30 seconds. A melting curve was performed to assess reaction products as follows: 95°C for 10 seconds, 65°C for 60 seconds and 97°C for 1 second. Data were expressed normalized to *rpoC*.

### RFP reporter assay

To quantify fluorescence produced by P_*gluD*_-PRS::mCherryOpt and P_*gluD*_-PRS^A70G^::mCherryOpt populations, fluorescence was monitored as described previously [40]. Overnight cultures in BHIS-Tm (500 μl) were collected by centrifugation and suspended in 30 μl 1X DPBS. Washed cells (20 μL) were added to 180 μl of 1X DPBS in a flat-bottom clear 96-well microtiter plate (Corning 3370) then removed from the anaerobic chamber. The starting cell density (OD_600_) was recorded, then the contents of each well were transferred to a flat-bottom black 96-well microtiter plate (VWR 76221–764) for fluorescence readings. Fluorescence was recorded at 30-minute intervals using a Biotek HT plate reader (excitation, 590 nm; emission, 645 nm; sensitivity setting, 100) with a continuous slow shake for three hours in aerobic conditions.

### Microscopy

Overnight cultures of the P_*gluD*_-PRS::mCherryOpt and P_*gluD*_-PRS^A70G^::mCherryOpt reporter strains were grown overnight in BHIS-Tm. Cultures were removed from the anaerobic chamber before a 500 μl aliquot of culture strain was combined with 120 μl fixative (20 μl 1M NaPO_4_, pH 7.4; 100 μl 16% paraformaldehyde) and incubated 30 minutes at room temperature followed by 60 minutes on ice [40,41]. Cells were washed with 1X DPBS before suspension in 500 μl 1X DPBS and aerobic incubation in the dark for two hours to allow for fluorophore maturation. Samples were applied to 1% agarose pads for microscopy using a Keyence BZ-X810 equipped with Chroma 49005-UF1 for RFP detection and a 60x oil immersion Nikon Plan Apo objective. Exposure and image capture settings were constant for all samples and all experiments. Four independent replicates of each strain were analyzed.

### Flow cytometry

Samples were grown and fixed as described for microscopy above. Cells washed with 1 mL of 1X DPBS, suspended in 500 μl 1X DPBS, and incubated in the dark aerobically for two hours to allow for mCherry fluorophore maturation. To detect *C*. *difficile* cells, the fixed samples were stained with the green fluorescent nucleic acid stain SYTO 9 (Invitrogen) to a final concentration of 1 μM for 20 minutes. Excess SYTO 9 was removed by washing with 1 mL 1X DPBS then resuspended in 1 mL 1X DPBS. Samples were then kept light-protected at 4°C until data acquisition.

Samples were run by the UNC Flow Cytometry Core using an Attune NxT (ThermoFisher Scientific) flow cytometer. A total of 100,000 events were collected for each sample. Data analysis was performed using FlowJo (Becton, Dickinson & Company) after single cells discrimination (see gating strategy, S3 Fig). mCherry positivity was determined by setting the baseline using WT bearing a PgluD-PRS^A70G^::mCherryOpt reporter mutant plasmid that is insensitive to c-di-GMP and lacks the expression of mCherry.

### Phenotype assays

Swimming motility: Overnight cultures (2 μL) were inoculated into 0.5X BHIS-0.3% agar medium [2]. Each plate contained all strains being compared. The diameter of growth was measured after 48 hours incubation at 37°C.

Surface motility: Overnight cultures (5 μL) were spotted on the surface of BHIS-1.8% agar-1% glucose medium [4,29]. Each plate contained all strains being compared. On day 6 post inoculation, diameters were determined by measuring the largest diameter of each colony and a second, perpendicular measurement, then averaging the values [4].

Biofilm formation: *O*vernight cultures were diluted 1:50 in BHIS, and 1 mL was added to 24-well untreated, flat-bottom plates (Corning) as four technical replicates. BHIS-only wells were included as controls. The plate was incubated statically for 24 hours, then removed from the anaerobic chamber for quantification of biofilm formation using crystal violet staining with all steps carried out at room temperature [29,30]. Culture supernatant was removed gently by pipetting, and remaining biomass was allowed to dry for 30 minutes. Biofilms were washed gently with 1 mL PBS, then stained with 750 μL 0.1% crystal violet (w/v in water) for 1 hour. The stain was removed, and each well was washed with 1 mL PBS. The stain was solubilized with 100% ethanol for 2 hours. Staining was measured by determining the absorbance at 570 nm, and values obtained from medium-only controls were subtracted. All strains were assayed in technical quadruplicate, which were averaged to obtain values for three independent biological replicates.

Vero cell rounding assay: 5 × 10^4^ Vero cells in 90 μL were seeded in each well of a tissue-culture treated, flat bottom 96-well plate (Corning) and incubated for 24 hours in a tissue culture incubator at 37°C and an atmosphere containing 5% CO_2_. *C*. *difficile* strains were grown in TY broth overnight (~16 hours). Bacterial cells were pelleted by centrifugation at 16,000 x g for 5 minutes. Supernatants were then sterilized by passing through a 0.45-μm filter. Samples were serial diluted in 1x DPBS and 10 μL were applied to each well with Vero cells. DPBS was used as a control. The cells were incubated overnight (~18 hours). Vero cell rounding was assessed using a 10x objective on a light microscope. Toxin titer was calculated as the reciprocal of the highest dilution that caused ≥80% cell rounding, log-transformed and normalized to OD_600_ of the starting overnight culture.

## Supporting information

S1 Table Strains and plasmids used in this study. (PDF)

S2 Table Oligonucleotides used in this study. (PDF)

S3 Table Data file. (XLSX)

S1 Fig Mapping of transcriptional start sites in or near the *pdcB* switch. Map of the transcriptional start sites (TSS) identified by 5’RACE in wild-type R20291 and Cdi2-ON::*phoZ*. Depicted are the sequences of the *pdcB* switch in the inverted/ON orientation (red) and published/OFF orientation (blue). The sequences corresponding to the inverted repeats are in bold text. TSS identified are indicated in green highlight. Putative -10 and -35 sequences in the promoters are underlined.
(TIF)

S2 Fig Kinetics of fluorescence development in PRS::mCherryOpt strains. Fluorescence produced by WT, *pdcB*Δ3-ON, and *pdcB*Δ3-OFF strains carrying the pP_*gluD*_-PRS::mCherryOpt plasmid was quantified over a 3-hour time course during which the fluorophore matures. R20291 with no plasmid and strains carrying pP_*gluD*_-PRS^A70G^::mCherryOpt, which encodes a riboswitch that is blind to c-di-GMP, were used as controls. Data are expressed as fluorescence units normalized to optical density, shown as the means and standard deviations for four biological replicates.
(TIF)

S3 Fig Quantification of percentage of the population that express mCherry by flow cytometry. (A) Gating strategy used to fit all samples. The gate quantifying the number of mCherry-positive cells in the population was set based on a negative control strain (WT bearing the c-di-GMP-blind PgluD-PRS^A70G^::mCherryOpt reporter). (B) Representative dot plots indicating the percentage of mCherry-positive cells in the population for WT, *pdcB*Δ3-ON, and *pdcB*Δ3-OFF strains carrying the pP_*gluD*_-PRS::mCherryOpt plasmid.
(TIF)

S4 Fig The impact of PdcB phase variation on toxin production is unclear. Toxin titers from supernatants grown overnight in TY broth for WT, Δ*pdcB*, *pdcB*Δ3-ON, and *pdcB*Δ3-OFF strains. *flg*Δ3-ON and *flg*Δ3-OFF locked strains were used as controls. Toxin titers were calculated as the reciprocal of the highest dilution that causes ≥80% rounding of Vero cells. Data are expressed after log-transformation and normalization to OD_600_ of the cultures. Means and standard deviation from 3 independent experiments each with 2 biological replicates are shown. **** *p* < 0.0001, *** *p* < 0.001, ** *p* < 0.01 by one-way ANOVA and Tukey’s post-test. Select statistical comparisons are shown.
(TIF)

S5 Fig The orientation of the *pdcC* switch controls *pdcC* expression. (A) Alkaline phosphatase (AP) assay using *C*. *difficile* strains with plasmid-borne transcriptional fusions to *phoZ*: the *pdcC* switch (Cdi3) in the published orientation (OFF), in the inverted orientation (ON), and two truncations of the 5’ end of the *pdcC* switch in the inverted orientation (ON). Promoterless *phoZ* was used as control. Means and standard deviations from 3 independent experiments are shown. **** *p* < 0.0001 by one-way ANOVA and Tukey’s post-test. (B) The transcriptional start site (TSS) identified by 5’RACE in R20291. The sequence of the *pdcC* switch in the inverted/ON orientation is in red and the published/OFF orientation is in blue. The sequences of the inverted repeats are in bold text. Green highlight indicates the TSS identified. Putative -10 and -35 sites are underlined.
(TIF)
