# Supplementary material for: Coordinated modulation of multiple processes through phase variation of a c-di-GMP phosphodiesterase in Clostridioides difficile
Source: PLoS Pathog. 2022 Jul 5;18(7):e1010677. doi: 10.1371/journal.ppat.1010677 (PMC9286219; doi:10.1371/journal.ppat.1010677)
Supplement: S1 Table — (PDF) [file ppat.1010677.s001.pdf]

Table S1. Strains and plasmids used in this study

***Clostridioides difficile* strains**			
**Lab Notation**	**Strain Name**	**Description**	**Reference**
RT273	*C. difficile* R20291	Ribotype 027 strain (Genbank Accession # FN545816)	(1)
RT1124	*C. difficile* 630	Ribotype 012 strain (Genbank Accession # AM180355)	(2)
RT1065	*C. difficile* UK1	Ribotype 027 strain	(3, 4)
RT1125	*C. difficile* VPI10463	Ribotype 003 strain	(5)
RT1357	*C. difficile* ATCC BAA 1875	Ribotype 078 strain	ATCC
RT1358	*C. difficile* ATCC 43598	Ribotype 017 strain	ATCC, (6)
RT1566	*sigD*	R20291 *sigD*::*ermB*	(7)
RT2622	Δ*pdcB*	R20291 Δ*pdcB* (in-frame deletion of CDR20291_0685)	This work
RT2797	*pdcB*Δ3-ON	R20291 with *pdcB* switch locked in the ON orientation by deletion of 3 nucleotides in the RIR	This work
RT2796	*pdcB*Δ3-OFF	R20291 with *pdcB* switch locked in the OFF orientation by deletion of 3 nucleotides in the RIR	This work
RT2816	WT pP$_{gluD}$-PRS::mCherryOpt	R20291 pDSW1728::P$_{gluD}$-PRS::mCherryOpt; fluorescent c-di-GMP reporter with *gluD* promoter, *pilA1* 5' UTR containing c-di-GMP riboswitch, and codon-optimized mCherry	This work
RT2813	WT pP$_{gluD}$-PRS^{A70G}::mCherryOpt	R20291 pDSW1728::P$_{gluD}$-PRS^{A70G}::mCherryOpt; mutation in riboswitch (A to G at position +70 in 5'UTR) rendering it blind to c-di-GMP	This work
RT2810	Δ*pdcB* pP$_{gluD}$-PRS::mCherryOpt	R20291 Δ*pdcB* pDSW1728::P$_{gluD}$-PRS::mCherryOpt; fluorescent c-di-GMP reporter with *gluD* promoter, *pilA1* c-di-GMP riboswitch, and codon-optimized mCherry	This work
RT2814	Δ*pdcB* pP$_{gluD}$-PRS^{A70G}::mCherryOpt	R20291 Δ*pdcB* pDSW1728::P$_{gluD}$-CdPRS^{A70G}::mCherryOpt; mutation in riboswitch (A to G at position +70 in 5'UTR) rendering reporter blind to c-di-GMP	This work
RT2812	*pdcB*Δ3-ON pP$_{gluD}$-PRS::mCherryOpt	R20291 *pdcB*-ON pDSW1728::P$_{gluD}$-CdPRS::mCherryOpt; fluorescent c-di-GMP reporter with *gluD* promoter, *pilA1* c-di-GMP riboswitch, and codon-optimized mCherry	This work
RT2815	*pdcB*Δ3-ON pP$_{gluD}$-PRS^{A70G}::mCherryOpt	R20291 *pdcB*-ON pDSW1728::P$_{gluD}$-CdPRS^{A70G}::mCherryOpt; mutation in riboswitch (A to G at position +70 in 5'UTR) rendering reporter blind to c-di-GMP	This work
RT2811	*pdcB*Δ3-OFF pP$_{gluD}$-PRS::mCherryOpt	R20291 *pdcB*-OFF pDSW1728::P$_{gluD}$-CdPRS::mCherryOpt; fluorescent c-di-GMP reporter with *gluD* promoter, *pilA1* c-di-GMP riboswitch, and codon-optimized mCherry	This work
RT2817	*pdcB*Δ3-OFF pP$_{gluD}$-PRS^{A70G}::mCherryOpt	R20291 *pdcB*-OFF pDSW1728::P$_{gluD}$-CdPRS^{A70G}::mCherryOpt; mutation in riboswitch (A to G at position +70 in 5'UTR) rendering it blind to c-di-GMP	This work
RT2195	WT pMC123::*phoZ*	R20291 pMC123::*phoZ;* promoterless negative control for alkaline phosphatase assay	This work
RT2095	WT pMC123::Cdi2-ONtrunc1::*phoZ*	R20291 pMC123::Cdi2-ONtrunc1::*phoZ;* Truncated version #1 of Cdi2 in the ON orientation fused to *phoZ*	This work
RT2096	WT pMC123::Cdi2-ONtrunc2::*phoZ*	R20291 pMC123::Cdi2-ONtrunc2::*phoZ;* Truncated version #2 of Cdi2 in the ON orientation fused to *phoZ*	This work
RT2097	WT pMC123::Cdi2-OFF::*phoZ*	R20291 pMC123::Cdi2-OFF::*phoZ*; Cdi2 in the OFF orientation fused to *phoZ*	This work
RT2098	WT pMC123::Cdi2-ON::*phoZ*	R20291 pMC123::Cdi2-ON::*phoZ*; Cdi2 in the ON orientation fused to *phoZ*	This work
RT2058	WT pMC123::Cdi3-ONtrunc1::*phoZ*	R20291 pMC123::Cdi3-ONtrunc1::*phoZ;* Truncated version #1 of Cdi3 in the ON orientation fused to *phoZ*	This work

RT2059	WT pMC123::Cdi3-ONtrunc2::*phoZ*	R20291 pMC123::Cdi3-ONtrunc2::*phoZ;* Truncated version #2 of Cdi3 in the ON orientation fused to *phoZ*	This work
RT2457	WT pMC123::Cdi3-OFF::*phoZ*	R20291 pMC123::Cdi3-OFF::*phoZ*; Cdi3 in the OFF orientation fused to *phoZ*	This work
RT2474	WT pMC123::Cdi3-ON::*phoZ*	R20291 pMC123::Cdi3-ON::*phoZ*; Cdi3 in the ON orientation fused to *phoZ*	This work
RT2621	Δ*pdcC*	R20291 Δ*pdcC* (in-frame deletion of CDR20291_1514)	This work
RT2839	WT pMC123::Cdi2-OFF(TSS1+2)::*phoZ*	R20291 pMC123::Cdi2-OFF(TSS1+2)::*phoZ*; Cdi2 in the OFF orientation that includes TSS1 and TSS2 fused to *phoZ*	This work
RT2960	WT pMC123::Cdi2-ON (TSS1+2)::*phoZ*	R20291 pMC123::Cdi2-ON(TSS1+2)::*phoZ*; Cdi2 in the ON orientation that includes TSS1 and TSS2 fused to *phoZ*	This work
RT2961	WT pMC123::Cdi2-ON (TSS1)::*phoZ*	R20291 pMC123::Cdi2-ON(TSS1)::*phoZ*; Cdi2 in the ON orientation that includes TSS1 only fused to *phoZ*	This work
RT2612	*flg*-Δ3 ON	R20291 with *flg* switch locked in the ON orientation by deletion of 3 nucleotides in the RIR	(8)
RT2609	*flg*-Δ3 OFF	R20291 with *flg* switch locked in the OFF orientation by deletion of 3 nucleotides in the RIR	(8)
***Escherichia coli* strains**			
**Lab Notation**	**Strain name**	**Description**	**Reference**
AC472	DH5α	*E. coli* F- φ80*lacZ*ΔM15 Δ(*lacZY*A-*argF*)U169 *recA1 endA1 hsdR*17(rκ -, mκ+) *phoA supE44 thi*-1 *gyrA96 relA1* λ- *tonA*	Invitrogen, (9)
RT270	HB101(pRK24)	*E. coli* used in conjugations with *C. difficile*, Ap^R	(10)
**Plasmids**			
**Lab notation**	**Plasmid name**	**Description**	**Reference**
pRT2460	pMSR0	*E. coli-C. difficile* shuttle vector for toxin/anti-toxin mediated allelic exchange	(11)
pRT2563	pMSR0::Δ*pdcB*	For in-frame deletion of *pdcB* (CDR20291_0685)	This work
pRT2795	pMSR0::*pdcB*Δ3-*ON*	For mutation in RIR to lock *pdcB* switch ON	This work
pRT2794	pMSR0::*pdcB*Δ3-*OFF*	For mutation in RIR to lock *pdcB* switch OFF	This work
pRT2766	pP$_{gluD}$-PRS::mCherryOpt	pDSW1728::P$_{gluD}$-PRS::mCherryOpt	This work
pRT2767	pP$_{gluD}$-PRS^{mut}::mCherryOpt	pDSW1728::P$_{gluD}$-PRS^{mut}::mCherryOpt	This work
pRT1343	pMC123::*phoZ*	Promoterless vector to use as negative control for alkaline phosphatase assay	(12)
pRT2017	pMC123::Cdi2-ONtrunc1::*phoZ*	Truncated version #1 of Cdi2 (*pdcB* switch) in the ON orientation fused to *phoZ*	This work
pRT2018	pMC123::Cdi2-Ontrunc2::*phoZ*	Truncated version #2 of Cdi2 (*pdcB* switch) in the ON orientation fused to *phoZ*	This work
pRT2019	pMC123::Cdi2-OFF::*phoZ*	Cdi2 (*pdcB* switch) in the OFF orientation that includes only TSS2 fused to *phoZ*	This work
pRT2020	pMC123::Cdi2-ON::*phoZ*	Cdi2 (*pdcB* switch) in the ON orientation that includes only TSS2 fused to *phoZ*	This work
pRT1643	pMC123::Cdi3-Ontrunc1::*phoZ*	Truncated version #1 of Cdi3 (*pdcC* switch) in the ON orientation fused to *phoZ*	This work
pRT1644	pMC123::Cdi3-Ontrunc2::*phoZ*	Truncated version #2 of Cdi3 (*pdcC* switch) in the ON orientation fused to *phoZ*	This work
pRT1963	pMC123::Cdi3-OFF::*phoZ*	Cdi3 (*pdcC* switch) in the OFF orientation fused to *phoZ*	This work

pRT1962	pMC123::Cdi3-ON::*phoZ*	Cdi3 (*pdcC* switch) in the ON orientation fused to *phoZ*	This work
pRT2562	pMSR0::Δ*pdcC*	For in-frame deletion of *pdcC* (CDR20291_1514)	This work
pRT942	pMC123::P_{gluD}-PRS::*pilA1*	Used to amplify the P_{gluD}-PRS	
pRT943	pMC123::P_{gluD}-PRS^{A70G}::*pilA1*	Used to amplify the P_{gluD}-PRS^{A70G}	
pRT2836	pMC123::Cdi2-OFF (TSS1+2)::*phoZ*	Cdi2 (*pdcB* switch) in the OFF orientation that includes both TSS1 and TSS2 fused to *phoZ*	This work
pRT2837	pMC123::Cdi2-ON (TSS1+2)::*phoZ*	Cdi2 (*pdcB* switch) in the ON orientation that includes both TSS1 and TSS2 fused to *phoZ*	This work
pRT2838	pMC123::Cdi2-ON (TSS1)::*phoZ*	Cdi2 (*pdcB* switch) in the ON orientation that includes only TSS1 to *phoZ*	This work

References

1. Stabler RA, He M, Dawson L, Martin M, Valiente E, Corton C, et al. Comparative genome and phenotypic analysis of *Clostridium difficile* 027 strains provides insight into the evolution of a hypervirulent bacterium. Genome Biology. 2009;10(9):R102.
2. Sebaihia M, Wren BW, Mullany P, Fairweather NF, Minton N, Stabler R, et al. The multidrug-resistant human pathogen *Clostridium difficile* has a highly mobile, mosaic genome. Nature Genetics. 2006;38(7):779-86.
3. Killgore G, Thompson A, Johnson S, Brazier J, Kuijper E, Pepin J, et al. Comparison of seven techniques for typing international epidemic strains of *Clostridium difficile*: restriction endonuclease analysis, pulsed-field gel electrophoresis, PCR-ribotyping, multilocus sequence typing, multilocus variable-number tandem-repeat analysis, amplified fragment length polymorphism, and surface layer protein A gene sequence typing. Journal of Clinical Microbiology. 2008;46(2):431-7.
4. Sorg JA, Sonenshein AL. Inhibiting the initiation of *Clostridium difficile* spore germination using analogs of chenodeoxycholic acid, a bile acid. Journal of Bacteriology. 2010;192(19):4983-90.
5. Sullivan NM, Pellett S, Wilkins TD. Purification and characterization of toxins A and B of *Clostridium difficile*. Infection Immunity. 1982;35(3):1032-40.
6. Depitre C, Delmee M, Avesani V, L'Haridon R, Roels A, Popoff M, et al. Serogroup F strains of *Clostridium difficile* produce toxin B but not toxin A. J Med Microbiol. 1993;38(6):434-41.
7. Anjuwon-Foster BR, Tamayo R. A genetic switch controls the production of flagella and toxins in *Clostridium difficile*. PLoS Genetics. 2017;13(3):e1006701.
8. Trzilova D, Warren MAH, Gadda NC, Williams CL, Tamayo R. Flagellum and toxin phase variation impacts intestinal colonization and disease development in a mouse model of *Clostridioides difficile* infection. Gut Microbes. 2022;14(1):2038854.
9. Hanahan D. Studies on transformation of *Escherichia coli* with plasmids. Journal of Molecular Biology. 1983;166(4):557-80.
10. McBride SM, Sonenshein AL. Identification of a genetic locus responsible for antimicrobial peptide resistance in *Clostridium difficile*. Infection and Immunity. 2011;79(1):167-76.
11. Peltier J, Hamiot A, Garneau JR, Boudry P, Maikova A, Hajnsdorf E, et al. Type I toxin-antitoxin systems contribute to the maintenance of mobile genetic elements in *Clostridioides difficile*. Communications Biology. 2020;3(1).
12. Garrett EM, Mehra A, Sekulovic O, Tamayo R. Multiple regulatory mechanisms control the production of CmrRST, an atypical signal transduction system in *Clostridioides difficile*. mBio. 2021;13(1):e0296921.
