# Supplementary material for: Coordinated modulation of multiple processes through phase variation of a c-di-GMP phosphodiesterase in Clostridioides difficile
Source: PLoS Pathog. 2022 Jul 5;18(7):e1010677. doi: 10.1371/journal.ppat.1010677 (PMC9286219; doi:10.1371/journal.ppat.1010677)
Supplement: S2 Table — (PDF) [file ppat.1010677.s002.pdf]

Table S2. Oligonucleotides used in this study.

Lab notation	Primer name	Target[a,b]	Sequence (5'- 3')
R2215	OS138	Cdi1 PUB	CGCAATTATTTGTTTTTCATATGGATAAAATTGG
R2216	OS139		GATTTTTATGTTAATGAATTGTTATAAAAAACATGG
R2213	OS140	Cdi1 INV	GGTAAGTTTGATTTTTATGTTAATGAATTG
R2214	OS141		CAGTTTGTGCACTAGCTATGCCTGC
R2262	OS101	Cdi2 PUB	CATTTCTAAGAAATATCCTAACATAAAAACAAAA
R2263	OS134		CGATTACACTACAGAATTAGAATGTCAATG
R2261	OS100	Cdi2 INV	GTTAAAAATTTAAGATATCTTTTCAGTATAATGGA
R2262	OS101		CATTTCTAAGAAATATCCTAACATAAAAACAAAA
R2267	OS106	Cdi3 INV	GATTTGTCGAAACCATTGTAATAAGA
R2268	OS107		CAATAGTTAAGACAATGAATATGCTACATTCT
R2268	OS107	Cdi3 PUB	CAATAGTTAAGACAATGAATATGCTACATTCT
R2269	OS136		GTAAATTCCTCATAAAAATTTCCTCCCA
R2175	qPCR_FlgSwit-ON	Cdi4 ON (PUB)	GTTTTCTTACCAAAGTGATACATTATTATATTAATG
R2177	qPCR_FlgSwit-REV		GCTATTGTCTGACTTCTTAAATTAGTTGCAT
R2176	qPCR_FlgSwit-OFF	Cdi4 OFF (INV)	CATTAATATAATAATGTATCACTTTGGTAAGAAAAC
R2177	qPCR_FlgSwit-REV		GCTATTGTCTGACTTCTTAAATTAGTTGCAT
R2264	OS104	Cdi5 PUB	GTAAATTAAGATGTATTTCATTTCTCAAAAATATCCT
R2266	OS135		GCTTTTATCGCAAGTTTGTTTTAAATGAC
R2264	OS104	Cdi5 INV	GTAAATTAAGATGTATTTCATTTCTCAAAAATATCCT
R2265	OS105		GTAAAGTTTATAAAATCTGAAAAGCTCAAGA
R2271	OS110	Cdi6 PUB	CTAGCCAATAGACAAGTTTCTAGAAAAATA
R2272	OS137		GAACAATTCTTGAATATTGTATTGAACATTAAGA
R2270	OS109	Cdi6 INV	GGAGATATATGGAGTTAGTGGTGCAA
R2271	OS110		CTAGCCAATAGACAAGTTTCTAGAAAAATA
R2378	OS196	Cdi7 PUB	GTACAGAAGTTACCCAGAAGCTTGT
R2379	OS197		TCCCCGCAATGGATGTTTTTTAATTCATC
R2378	OS196	Cdi7 INV	GTACAGAAGTTACCCAGAAGCTTGT
R2380	OS198		TCCCAATTTAAATGTAGAGGTCATCAAT
R2273	OS142	*rpoA*	TCATTACCAGGTGTAGCAGTGAATGC
R2274	OS143		TGATAGAGCATGGTCCTTGAGCTTCT
R3082	CDR0685_F1	Δ*pdcB*	CATTGATTTCTTTCAGTTTCGGATCCGTAACCCTTAGTTGTAAAAGGGTTC
R3083	CDR0685_R1		CACTTTGATAGTTGGTCTAAACTTAAAGAGTTCGATTCTTCTC
R3084	CDR0685_F2		CTCTTTAAGTTTAGACCAACTATCAAAGTGATGTACATAAAATGAG
R3085	CDR0685_R2		GACGTCGACTCTAGAGGATCCCAGAACATTCCACGGTAAAATGG
R3086	CDR0685IE_F1	*pdcB*Δ3-OFF and *pdcB*Δ3-ON	CATTGATTTCTTTCAGTTTCGGATCCGTAATTGGTATACTTCCCTAGTTTACG
R3089	CDR0685IE_R2		GACGTCGACTCTAGAGGATCCCATCTTTCCATATTTGAACATCATCC
R3090	CDR0685IEpub_R1	*pdcB*Δ3-OFF	GTAAAGTTACTATTTATTGAAAATTTAGATAC
R3091	CDR0685IEpub_F2		CTAAATTTTCAATAAATAGTAACTTTACAAC
R3092	CDR0685IEinv_R1	*pdcB*Δ3-ON	GTAAGGTTCTTTTTTTTTATAATAAAATAGC
R3093	CDR0685IEinv_F2		GCTATTTTATTATAAAAAAAAAGAACCTTAC
R3094	phoZ-GSP1	5'RACE for *pdcB* TSS2	CATCTTCTGGATAAGTG
R3095	phoZ-GSP2		GCTTGCTGTCCGACCAAATAGGTATC
R3137	GSP1_short-pdcB	5'RACE for *pdcB* TSS1	GGTACATTTTTAGTACATG
R3138	GSP2_short-pdcB		GAGTTGCTTACAGGGTATCTAGGATTGATG

R2419	0685inv_trunc1	Cdi2::*phoZ* fusions	ATGCAGAATTCCTTAATTTGATTTGATATGTATATTTTTATAGC
R2420	0685inv_trunc2		ATGCAGAATTCGTTAAATTTATGAACATTTTTTGTTTTTATG
R2421	0685pub-LIR		ATGCAGAATTCCTTTTTTTTTATAATAAAATAGCTATAAAAATATAC
R2422	0685inv-LIR		ATGCAGAATTCCTATTTATTGAAAATTTAGATACTTTTCT
R2330	0685_pubR		ATGCAGGATCCCGTACATAGTTTCCATTTGTTGTAAA
R3139	R20291_0685qF	*pdcU* qRT-PCR	CCGGATGATGTTCAAATATGGAAAG
R3140	R20291_0685qR		TGGGTCATCCGACACATAAAC
R850	rpoCqF	*rpoC* qRT-PCR	CTAGCTGCTCCTATGTCTCACATC
R851	rpoCqR		CCAGTCTCTCCTGGATCAACTA
R3063	PgluD-CdPRS Gib	c-di-GMP biosensor	GAATTCTGCATCAAGCTAGCGAAAAGGAAATAATAGGAATATGGTAG
R3064	PgluD-CdPRS Gib		CTTTACTGCAGGAGCTCACTTACTTATAATATATCTGGTTAAAAATCTAAG
R3141	CDR1514_F1	Δ*pdcC*	CATTGATTTCTTTCAGTTTCGGATCCGTCTATCAAACTCGCTATAATATGTAGTG
R3142	CDR1514_R1		CTTACCAAGTTGGACCTCATAAAAATTTCCTCCCATTAAAAC
R3143	CDR1514_F2		GAAATTTTTATGAGGTCCAACTTGGTAAGAGGGTAATTAATCTG
R3144	CDR1514_R2		GACGTCGACTCTAGAGGATCCGTCAACACCACCTATAGGTTCATC
R2218	pdcCIE_pubF	Cdi3::*phoZ* fusions	ATGGAGCTCAATAAAATTTTTCAGACAATTCAAACAAAAATAATC
R2219	pdcCIE-invF		ATGGAGCTCTATCTACTTTAATTTTGTAAAATTTTCTGCTAC
R2220	pdcCIE-R		ATGGGTACCGCAAATAAGTACATCTCAAAAGTTTCC
R1977	pdcC_IE_truncated1R		TACGTGAATTCATTTATTAAAAGGTGATTATTTTTG
R1978	pdcC_IE_truncated2R		TACGTGAATTCGCATATTCATTGTCTTAACTATTG
R3096	CDR0685_F0	To screen for Δ*pdcB* allele	GCAAGAACCAATCAGTTACTTGAAG
R3097	CDR0685_R0		GCTATGGAACATCCAGAAGAATATCC
R3146	CDR1514_F0	To screen for Δ*pdcC* allele	GCTTTATTACCAGCTATATGTGAAGAAC
R3147	CDR1514_R0		CACTTATTAAGTGGGTAGCAATCAG
R837	pUC19mcsF	To screen for pMC123 plasmid	TCTTCGCTATTACGCCAG
R839	m13R		AACAGCTATGACCATG
R3148	GSP1_pdcC	5'RACE for *pdcC* TSS	GACTCAAAGTTATTTATGG
R3149	GSP2_pdcC		GAGTTTGCATAAAGAATAGACTGGTGTTGTG
R3108	TSS1_phoZ F	Cdi2(TSS1)::*phoZ* fusions	CGTTGTAAAACGACGGCCAGTGAATTCGGATTAAGTGTAATAAAATAGAAAACAAG
R3109	TSS1_phoZ R		CTTCATATCTACCCATACATTGACGGATCCCGTACATAGTTTCCATTTGTTG
R3110	TSS1_phoZ_truncON R		CTTCATATCTACCCATACATTGACGGATCCATGTTCATAAATTTAACTATTAACATAG

[a] PUB indicates the intended template corresponds to that in the R20291 reference genome (FN585816); INV indicates the intended template has the inverted switch sequence

[b] Restriction sites are underlined
