# Supplementary figures and images for: Coordinated modulation of multiple processes through phase variation of a c-di-GMP phosphodiesterase in Clostridioides difficile
Source: PLoS Pathog. 2022 Jul 5;18(7):e1010677. doi: 10.1371/journal.ppat.1010677 (PMC9286219; doi:10.1371/journal.ppat.1010677)

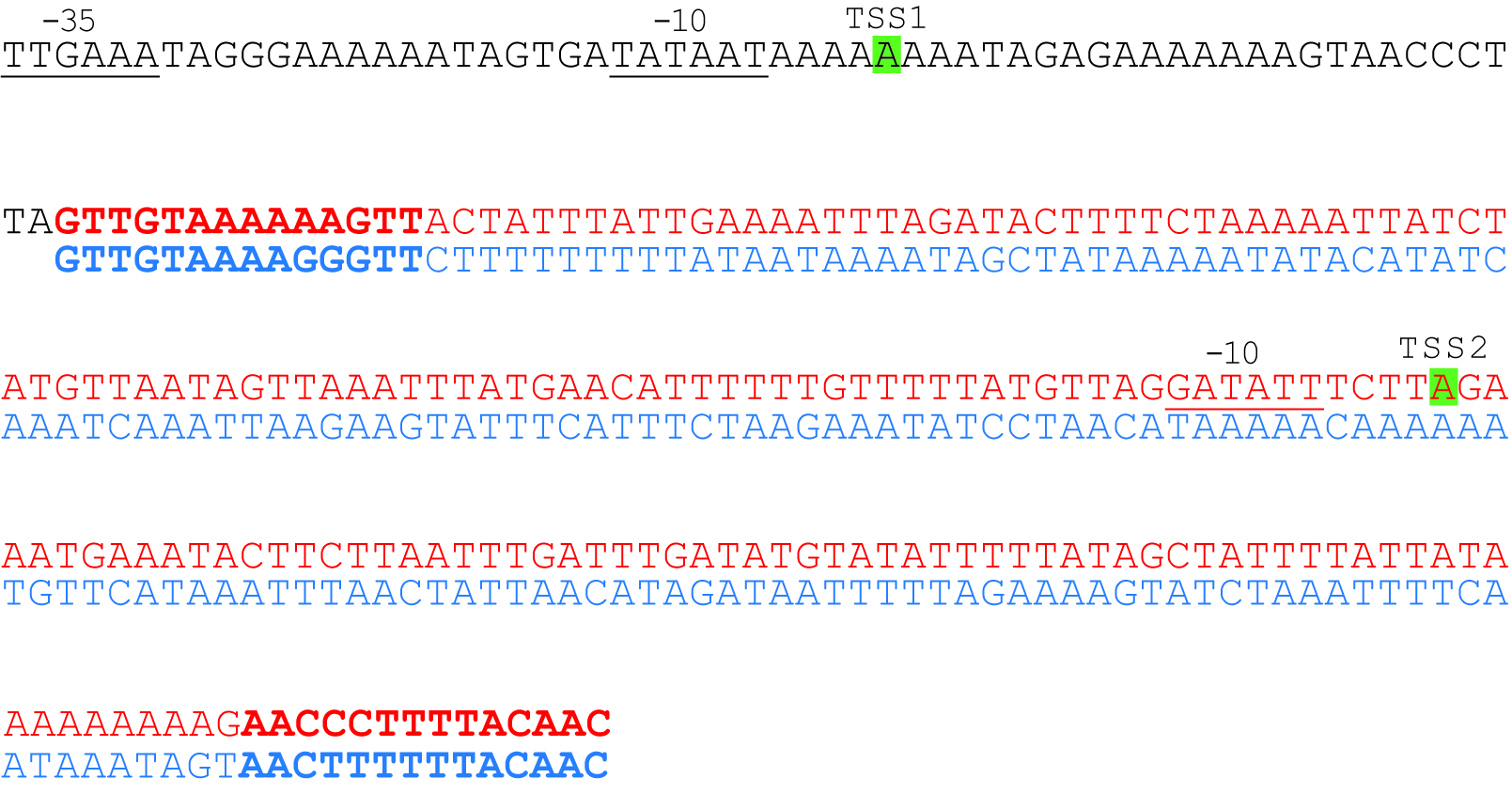

Supplement: S1 Fig — Map of the transcriptional start sites (TSS) identified by 5’RACE in wild-type R20291 and Cdi2-ON::phoZ. Depicted are the sequences of the pdcB switch in the inverted/ON orientation (red) and published/OFF orientation (blue). The sequences corresponding to the inverted repeats are in bold text. TSS identified are indicated in green highlight. Putative -10 and -35 sequences in the promoters are underlined. (TIF) [file ppat.1010677.s004.tif]

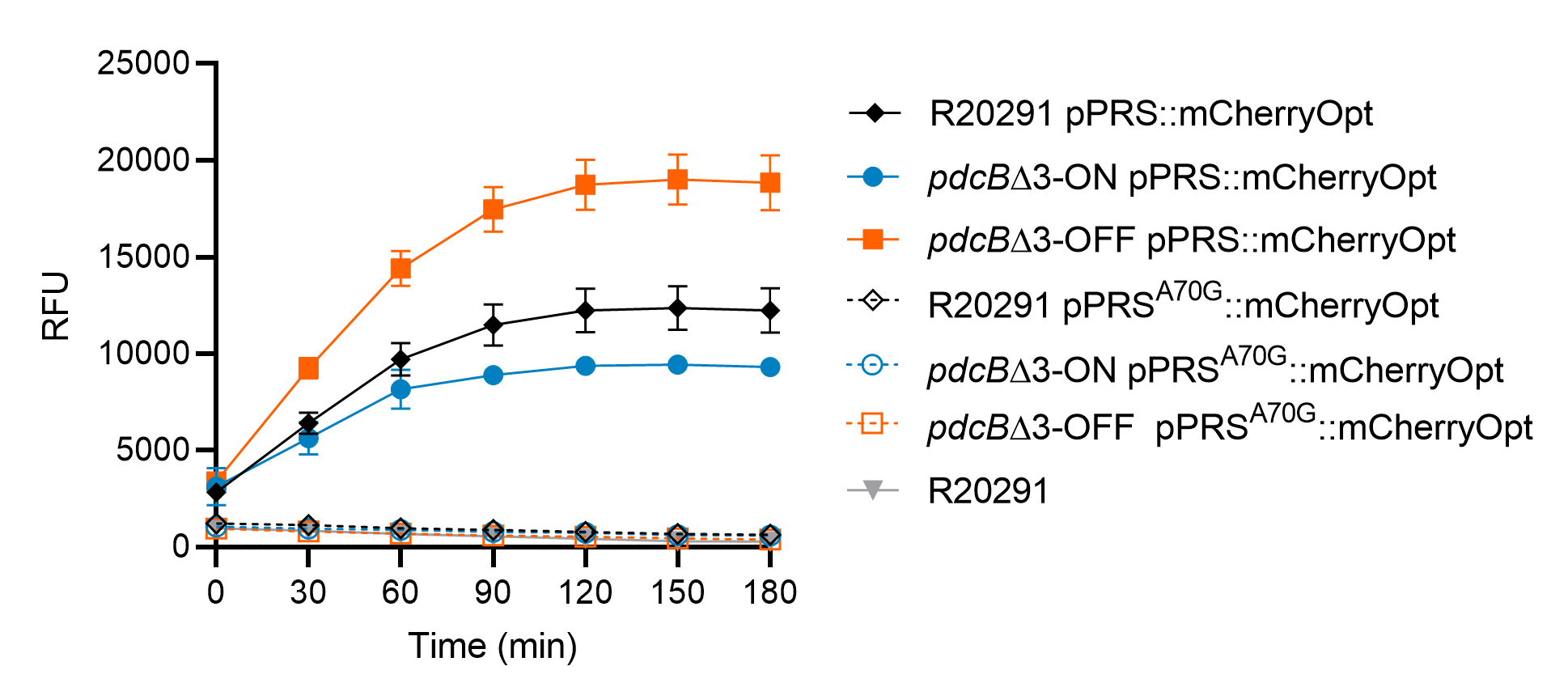

Supplement: S2 Fig — Fluorescence produced by WT, pdcBΔ3-ON, and pdcBΔ3-OFF strains carrying the pPgluD-PRS::mCherryOpt plasmid was quantified over a 3-hour time course during which the fluorophore matures. R20291 with no plasmid and strains carrying pPgluD-PRSA70G::mCherryOpt, which encodes a riboswitch that is blind to c-di-GMP, were used as controls. Data are expressed as fluorescence units normalized to optical density, shown as the means and standard deviations for four biological replicates. (TIF) [file ppat.1010677.s005.tif]

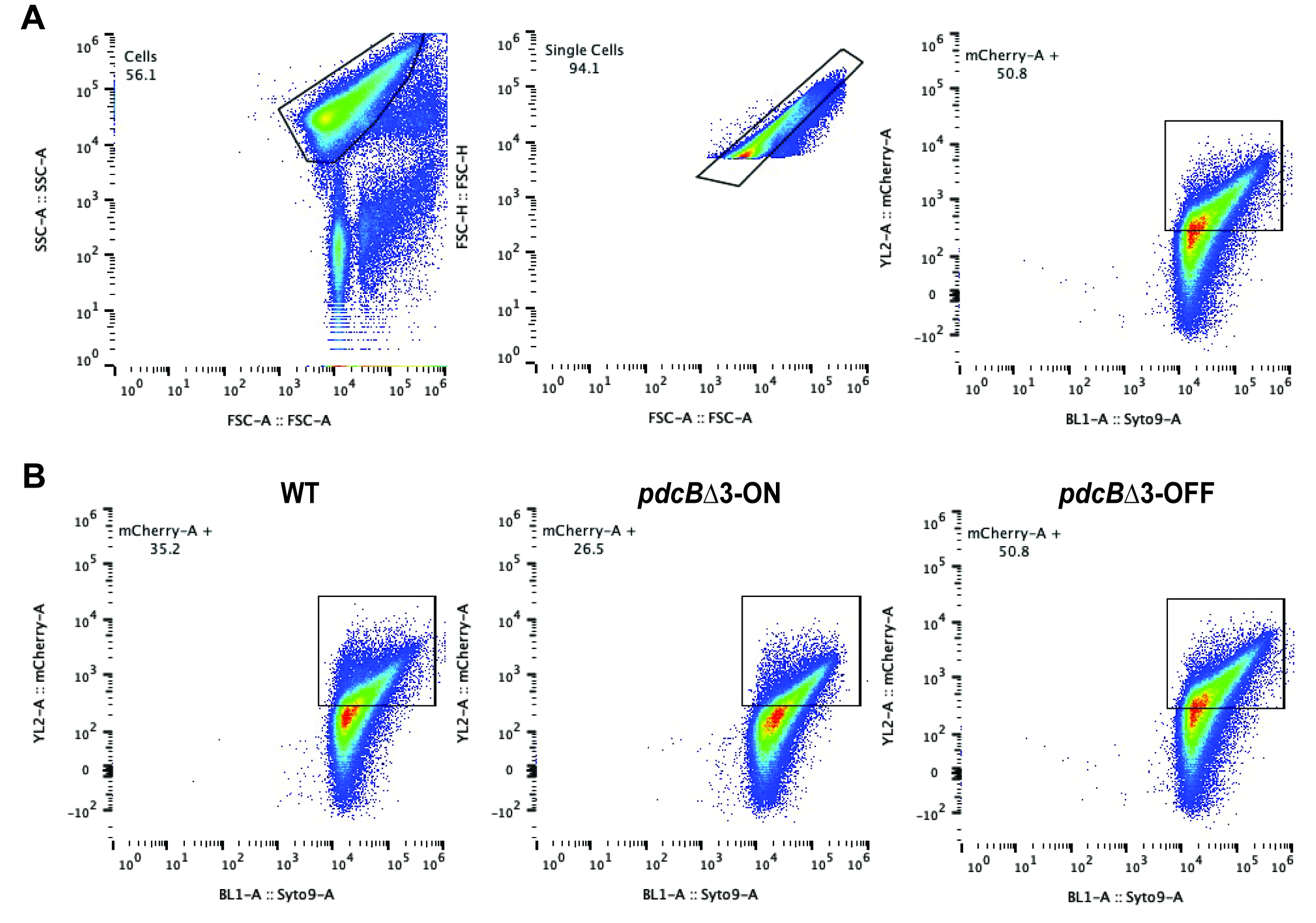

Supplement: S3 Fig — (A) Gating strategy used to fit all samples. The gate quantifying the number of mCherry-positive cells in the population was set based on a negative control strain (WT bearing the c-di-GMP-blind PgluD-PRSA70G::mCherryOpt reporter). (B) Representative dot plots indicating the percentage of mCherry-positive cells in the population for WT, pdcBΔ3-ON, and pdcBΔ3-OFF strains carrying the pPgluD-PRS::mCherryOpt plasmid. (TIF) [file ppat.1010677.s006.tif]

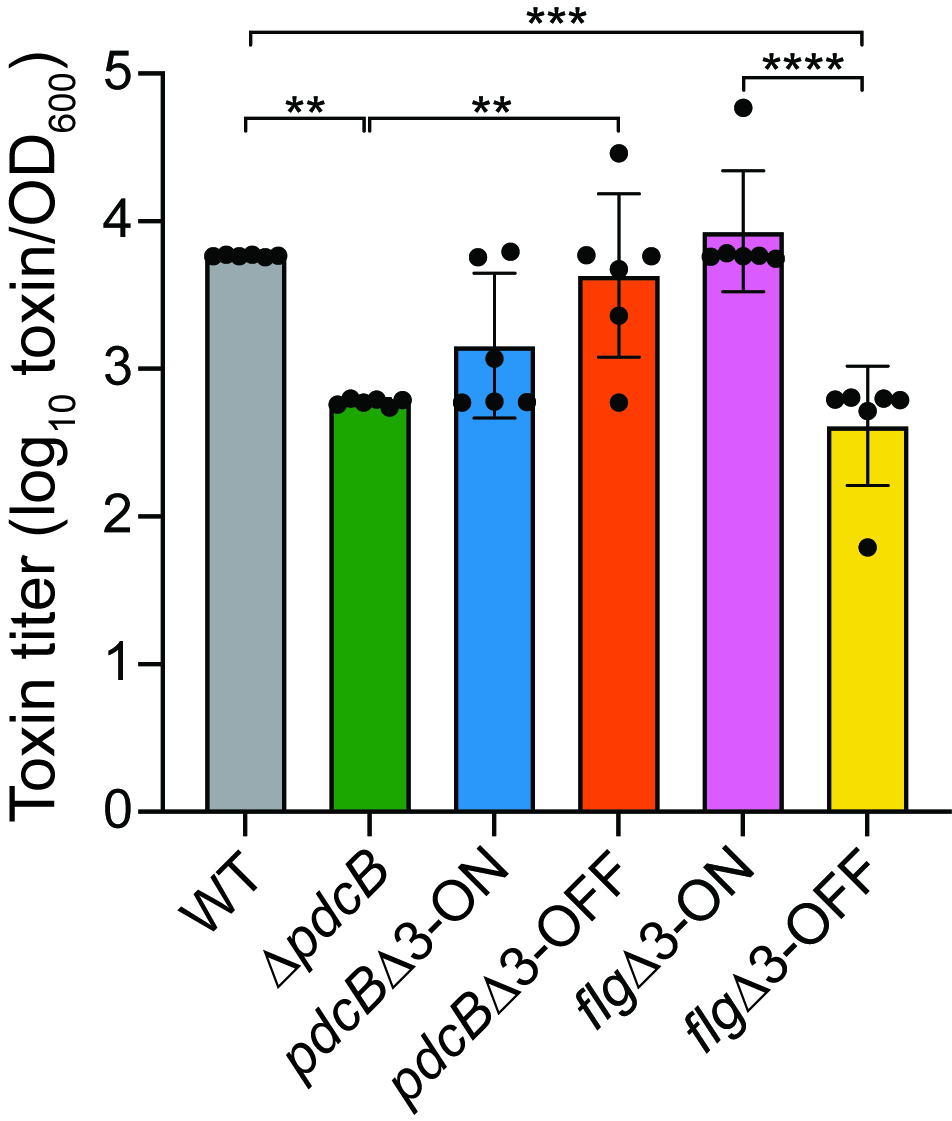

Supplement: S4 Fig — Toxin titers from supernatants grown overnight in TY broth for WT, ΔpdcB, pdcBΔ3-ON, and pdcBΔ3-OFF strains. flgΔ3-ON and flgΔ3-OFF locked strains were used as controls. Toxin titers were calculated as the reciprocal of the highest dilution that causes ≥80% rounding of Vero cells. Data are expressed after log-transformation and normalization to OD600 of the cultures. Means and standard deviation from 3 independent experiments each with 2 biological replicates are shown. **** p < 0.0001, *** p < 0.001, ** p < 0.01 by one-way ANOVA and Tukey’s post-test. Select statistical comparisons are shown. (TIF) [file ppat.1010677.s007.tif]
